# Supplementary material for: Non-structural carbohydrates mediate seasonal water stress across Amazon forests
Source: Nat Commun. 2021 Apr 19;12:2310. doi: 10.1038/s41467-021-22378-8 (PMC8055652; doi:10.1038/s41467-021-22378-8)
Supplement: Supplementary file 1 — Supplementary Information [file 41467_2021_22378_MOESM1_ESM.pdf]

SUPPLEMENTARY INFORMATION

Non-structural carbohydrates mediate seasonal water stress across Amazon forests

Caroline Signori-Müller[1,2*], Rafael S. Oliveira[3], Fernanda de Vasconcellos Barros[4,5], Julia Valentim Tavares[2], Martin Gilpin[2], Francisco Carvalho Diniz[2], Manuel J. Marca Zevallos[6,7], Carlos A. Salas Yupayccana[7], Martin Acosta[8], Jean Bacca[6], Rudi S. Cruz Chino[7], Gina M. Aramayo Cuellar[9], Edwin R. M. Cumapa[6], Franklin Martinez[9], Flor M. Pérez Mullisaca[6], Alex Nina[7], Jesus M. Bañon Sanchez[6], Leticia Fernandes da Silva[8], Ligia Tello[6], José Sanchez Tintaya[6], Maira T. Martinez Ugarteche[9], Timothy R. Baker[2], Paulo R.L. Bittencourt[4,5], Laura S. Borma[10], Mauro Brum[11,5], Wendeson Castro[8], Eurídice N. Honorio Coronado[12], Eric G. Cosio[13], Ted R. Feldpausch[4], Letícia d'Agosto Miguel Fonseca[10], Emanuel Gloor[2], Gerardo Flores Llampazo[14], Yadvinder Malhi[15], Abel Monteagudo Mendoza[6], Victor Chama Moscoso[6], Alejandro Araujo-Murakami[9], Oliver L. Phillips[2], Norma Salinas[15,13], Marcos Silveira[8], Joey Talbot[16], Rodolfo Vasquez[17], Maurizio Mencuccini[18,19] and David Galbraith[2].

[1] Department of Plant Biology, Institute of Biology, Programa de Pós Graduação em Biologia Vegetal, University of Campinas, Campinas, Brazil

[2] School of Geography, University of Leeds, Leeds, UK

[3] Department of Plant Biology, Institute of Biology, University of Campinas, Campinas, Brazil

[4] Geography, College of Life and Environmental Sciences, University of Exeter, Exeter, UK

[5] Department of Plant Biology, Institute of Biology, Programa de Pós Graduação em Ecologia, University of Campinas, Campinas, Brazil

[6] Universidad Nacional de San Antonio Abad del Cusco, Cusco, Peru

[7] Pontificia Universidad Católica del Perú, Lima, Perú

[8] Programa de Pós-Graduação em Ecologia e Manejo de Recursos Naturais, Universidade Federal do Acre, Rio Branco, Brazil

[9] Museo de Historia Natural Noel Kempff Mercado, Universidad Autonoma Gabriel Rene Moreno, Santa Cruz, Bolivia[4]

[10] Earth System Science Centre, National Institute for Space Research, São José dos Campos, Brazil

[11] Department of Ecology and Evolutionary Biology, University of Arizona, Tucson, USA

[12] Instituto de Investigaciones de la Amazonia Peruana, Iquitos, Peru

[13] Sección Química, Pontificia Universidad Católica del Perú, Lima, Peru

[14] Universidad Nacional Jorge Basadre de Grohmann, Tacna, Peru

[15] Environmental Change Institute, School of Geography and the Environment, University of Oxford, Oxford, UK

[16] Institute for Transport Studies, University of Leeds, Leeds, UK

[17] Jardín Botánico de Missouri, Pasco, Peru

[18] CREAF, Campus UAB, Cerdanyola del Vallés, Spain

[19] ICREA, Barcelona, Spain

*carol.signori@gmail.com

SUPPLEMENTARY NOTES

Supplementary Note 1

Abstract in Portuguese | Resumo em Português

Carboidratos não-estruturais mediam o estresse hídrico sazonal de florestas Amazônicas

Os carboidratos não estruturais (CNE) são os substratos mais importantes para o metabolismo das plantas e estão envolvidos na mediação das respostas das plantas à seca. Apesar da sua importância, a dinâmica dos CNE em florestas tropicais ainda é pouco estudada. Nesse trabalho, nós apresentamos dados de CNE de folhas e ramos de 82 espécies de árvores de dossel da Amazônia, amostradas em seis sítios abrangendo um amplo gradiente de precipitação. Durante a estação chuvosa, as concentrações totais de CNE (CNE_T) em ambos os órgãos foram marcadamente similares ao longo dos sítios. No entanto, os CNE_T e suas frações, açúcares solúveis (AS) e amido, variaram muito mais ao longo dos sítios durante a estação seca. Notavelmente, a proporção dos CNE_T foliares na forma de AS aumentou consideravelmente na estação seca em quase todas as espécies nos sítios mais secos, indicando um importante papel dos AS na mediação do estresse hídrico nesses sítios. Esse ajuste no balanço dos CNE_T nas folhas não foi observado em espécies de árvores menos adaptadas ao déficit hídrico, mesmo durante condições de seca excepcional. Assim, a dinâmica do carbono nas folhas pode ajudar a explicar a composição florística ao longo de gradientes de disponibilidade hídrica na Amazônia, e melhorar as predições das respostas dessas florestas às futuras mudanças climáticas.

Supplementary Note 2

Abstract in Spanish | Resumen en Español

Carbohidratos no estructurales median el estrés hídrico estacional de los bosques Amazónicos

Los carbohidratos no estructurales (CNE) son los principales sustratos para el metabolismo de las plantas y han estado implicados como intermediarios en la mortalidad inducida por la sequía. A pesar de su importancia, la dinámica de los CNE en los bosques tropicales sigue siendo poco conocida. En este estudio, presentamos datos de CNE de hojas y ramas para 82 especies de árboles del dosel de la Amazonía, recogida en seis localidades que abarcan un amplio gradiente de precipitación. Durante la temporada de lluvias, las concentraciones totales de los CNE (CNE_T) en ambos órganos fueron notablemente similares en todos los sitios. Sin embargo, los CNE_T y sus componentes, azúcares solubles (AS) y almidón, variaron mucho más entre sitios durante la estación seca. En particular, la proporción de los CNE_T foliares en forma de AS aumentó mucho en la estación seca en casi todas las especies en los sitios más secos, lo que implica un papel importante de los AS en la mediación del estrés hídrico en estos sitios. Este ajuste del balance de los CNE_T de las hojas no se observó en especies de árboles menos adaptadas al déficit hídrico, incluso en condiciones excepcionales de sequía. Por lo tanto, el metabolismo del carbono de las hojas puede ayudar a explicar la composición florística a lo largo de los gradientes de disponibilidad hídrica en la Amazonia y mejorar las predicciones de las respuestas de los bosques al cambio climático futuro.

SUPPLEMENTARY FIGURES

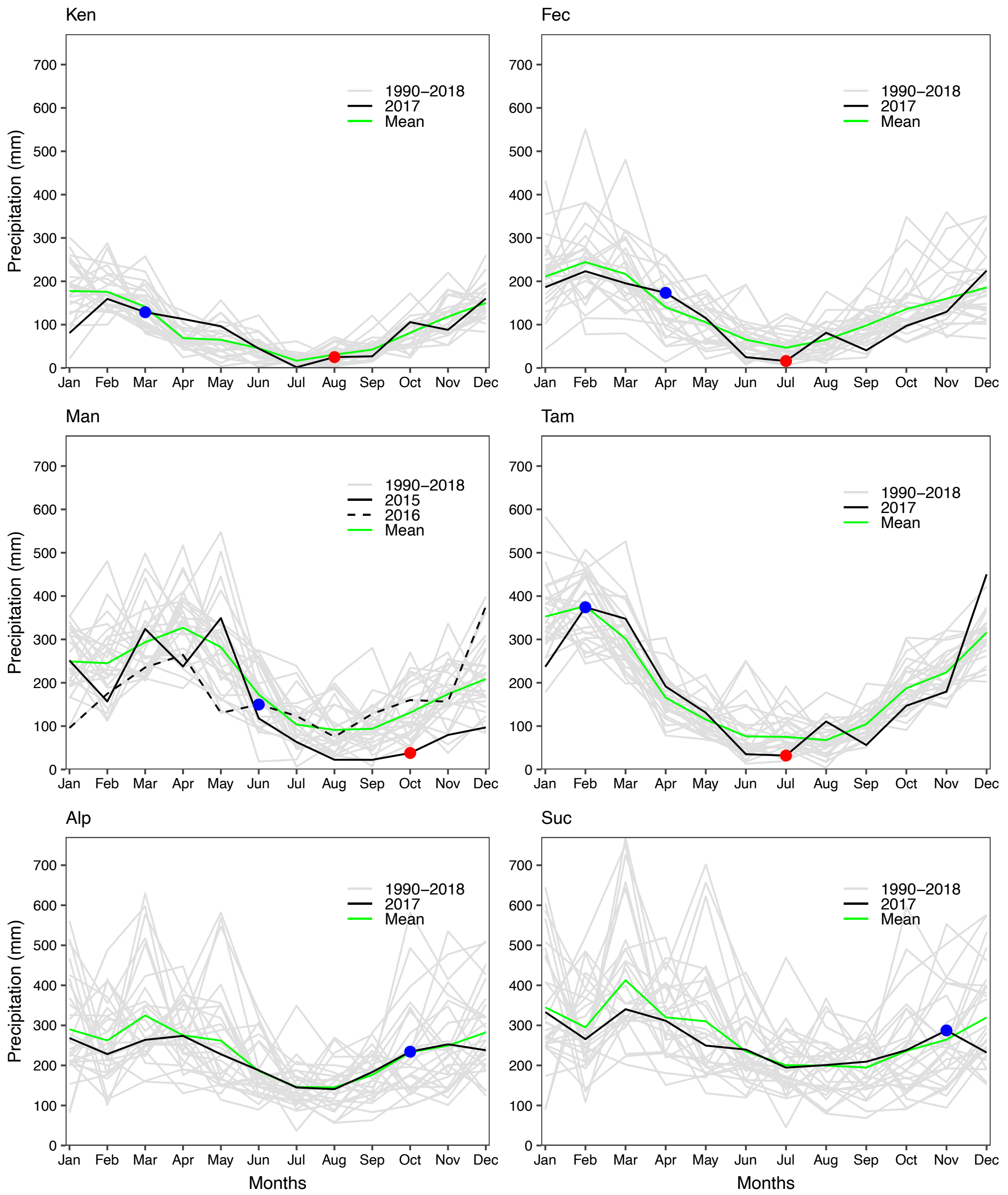

Supplementary Fig. 1 | Monthly precipitation at the time of NSC and Ψ_{MD} sampling at each site. Grey lines represent the precipitation from 1990 to 2018; the green line represents the mean precipitation for the period of 1990 to 2018; and black lines the precipitation in the years we collected the samples. Blue and red dots denotate the sampling month for wet and dry season respectively. To construct these figures we used CRU-TS 4.03[1] downscaled with WorldClim 2.1[2]; spatial resolution 2.5 minutes.

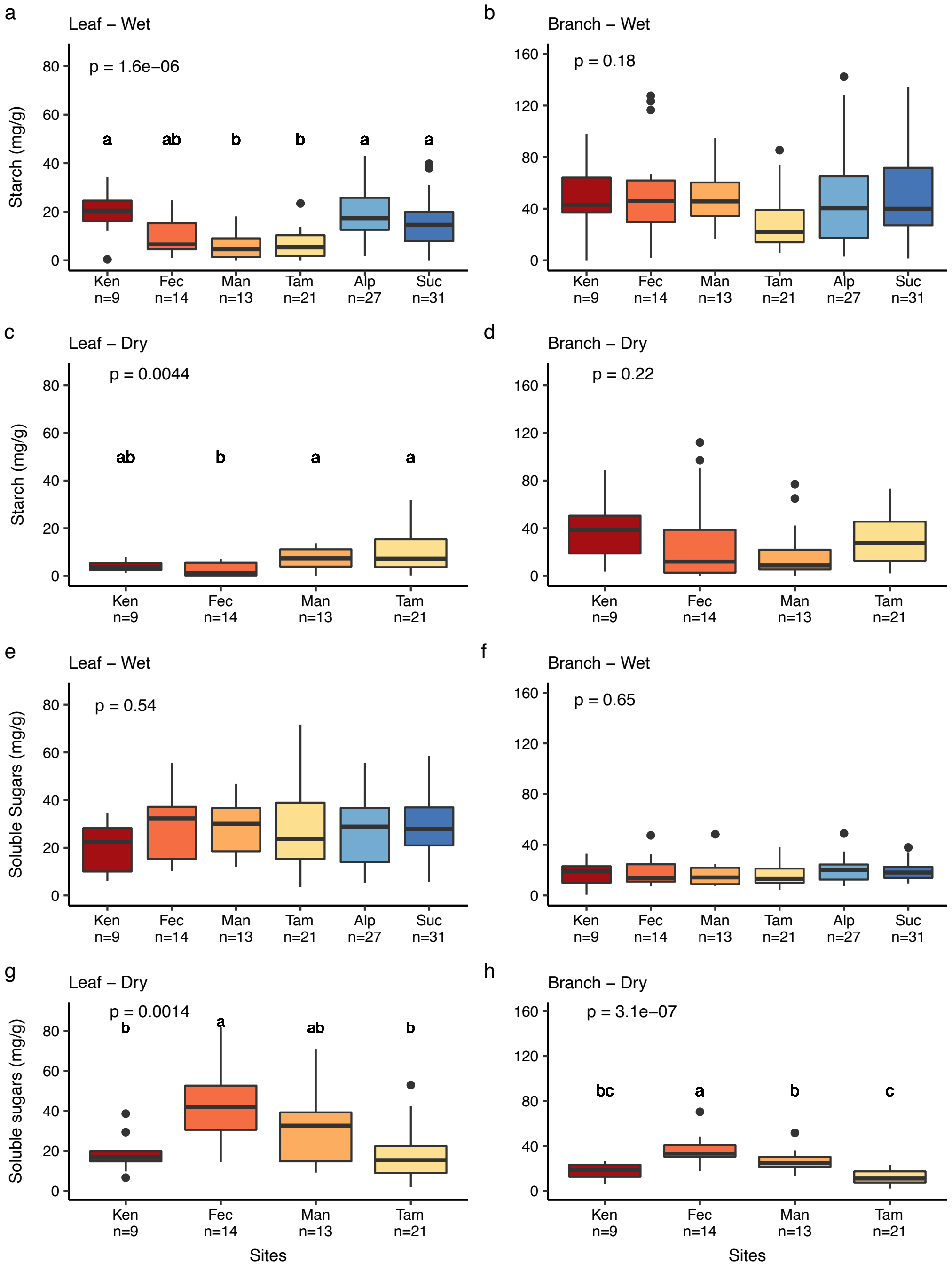

Supplementary Fig. 2 | Starch and soluble sugar (SS) concentrations across sites during the wet and dry seasons. Boxplots were constructed based on mean species values at each site. Concentrations of starch and SS are displayed for leaves (panels a, c, e, g) and branches (b, d, f, h). Each box encompasses the 25th to 75th percentiles; the median is indicated by the horizontal line in each box while external horizontal lines indicate the 10th and 90th percentiles; dots indicate outliers. Sites are ordered and colour-coded from left to right from driest to wettest; red to yellow boxes represent the seasonal sites and two blue boxes the aseasonal sites. n indicate the number of species sampled in each site. Differences among sites were tested using one-way Kruskal-Wallis. Sites with different letters are statistically distinguishable ($p < 0.05$, post-hoc Mann-Whitney-Wilcoxon Rank Sum test using Bonferroni correction is indicated by small letters).

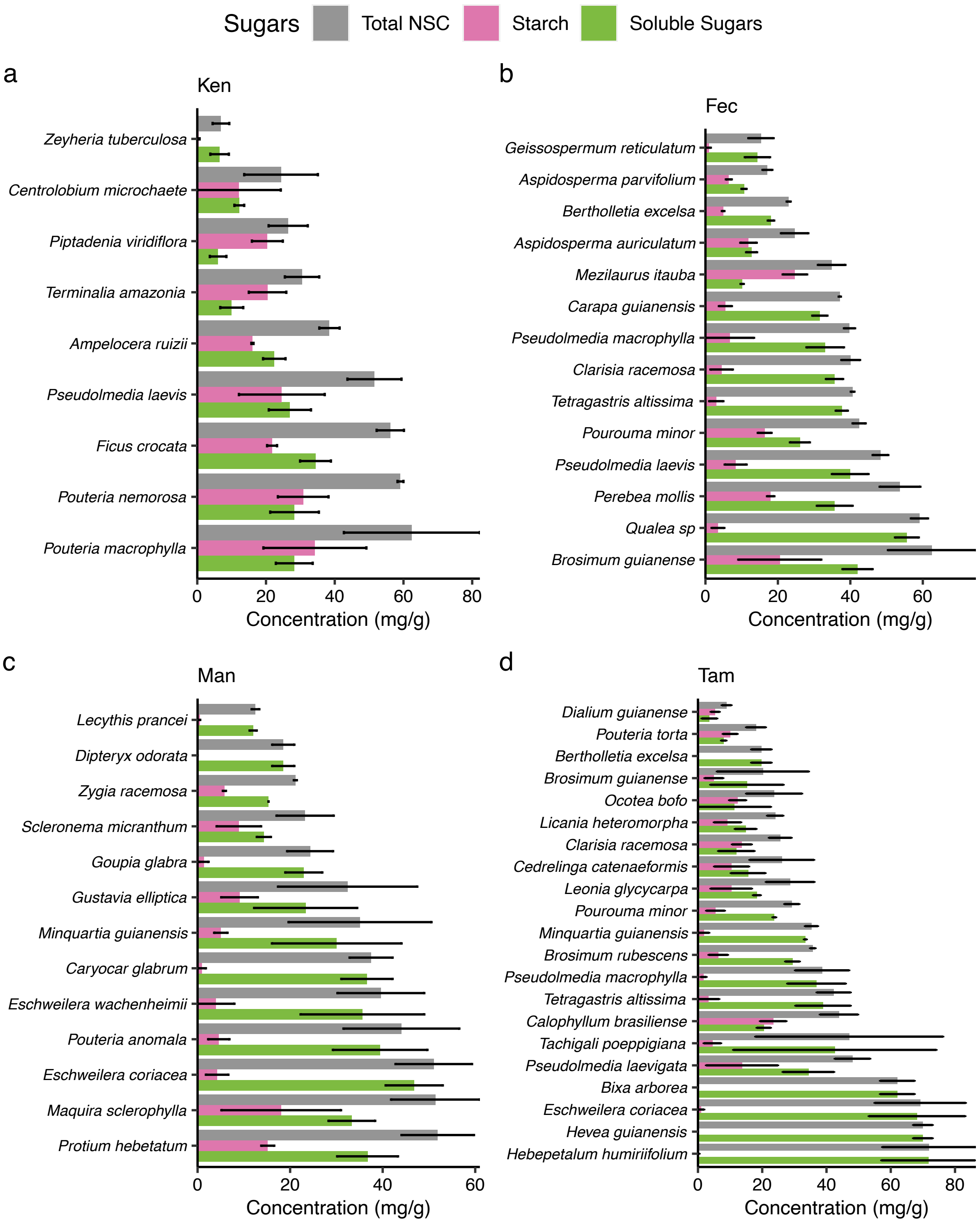

Sugars
Total NSC
Starch
Soluble Sugars
a
Ken
Zeyheria tuberculosa
Centrolobium microchaete
Piptadenia viridiflora
Terminalia amazonia
Ampelocera ruizii
Pseudolmedia laevis
Ficus crocata
Pouteria nemorosa
Pouteria macrophylla
0
20
40
60
80
Concentration (mg/g)
b
Fec
Geissospermum reticulatum
Aspidosperma parvifolium
Bertholletia excelsa
Aspidosperma auriculatum
Mezilaurus itauba
Carapa guianensis
Pseudolmedia macrophylla
Clarisia racemosa
Tetragastris altissima
Pourouma minor
Pseudolmedia laevis
Perebea mollis
Qualea sp
Brosimum guianense
0
20
40
60
Concentration (mg/g)
c
Man
Lecythis prancei
Dipteryx odorata
Zygia racemosa
Scleronema micranthum
Goupia glabra
Gustavia elliptica
Minquartia guianensis
Caryocar glabrum
Eschweilera wachenheimii
Pouteria anomala
Eschweilera coriacea
Maquira sclerophylla
Protium hebetatum
0
20
40
60
Concentration (mg/g)
d
Tam
Dialium guianense
Pouteria torta
Bertholletia excelsa
Brosimum guianense
Ocotea bofo
Licania heteromorpha
Clarisia racemosa
Cedrelinga catenaeformis
Leonia glycycarpa
Pourouma minor
Minquartia guianensis
Brosimum rubescens
Pseudolmedia macrophylla
Tetragastris altissima
Calophyllum brasiliense
Tachigali poeppigiana
Pseudolmedia laevigata
Bixa arborea
Eschweilera coriacea
Hevea guianensis
Hebepetalum humiriifolium
0
20
40
60
80
Concentration (mg/g)

continue…

…continuation

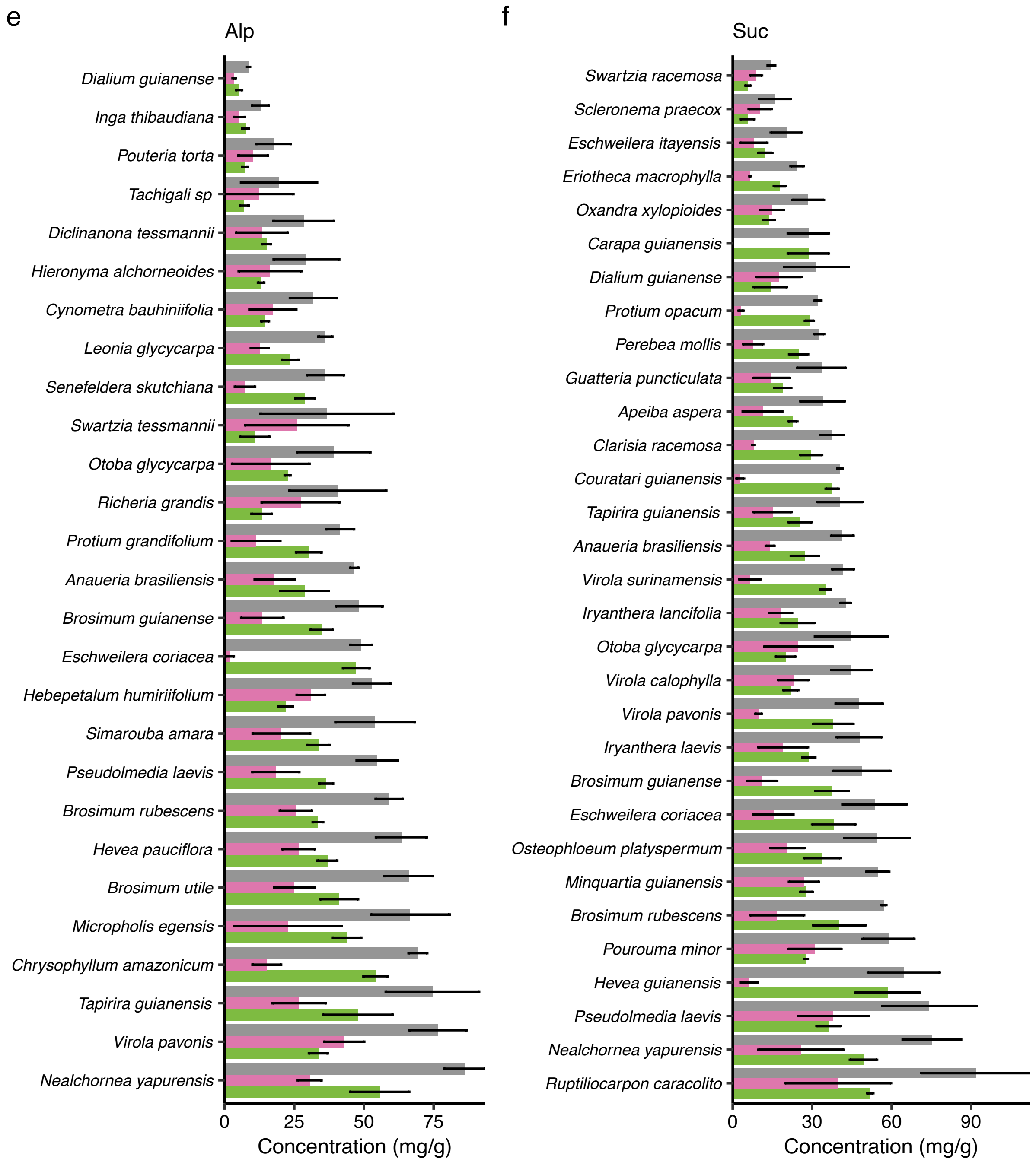

Supplementary Fig. 3 | Wet season mean leaf total non-structural carbohydrates (NSC_T), starch and soluble sugar (SS) concentrations in all species of each site. Each panel represent one site, as follow: a) Ken, b) Fec, c) Man, d) Tam, e) Alp and f) Suc. Colours represent NSC_T (grey), starch (pink) and SS (green). Horizontal black bars denotate one standard error of the mean.

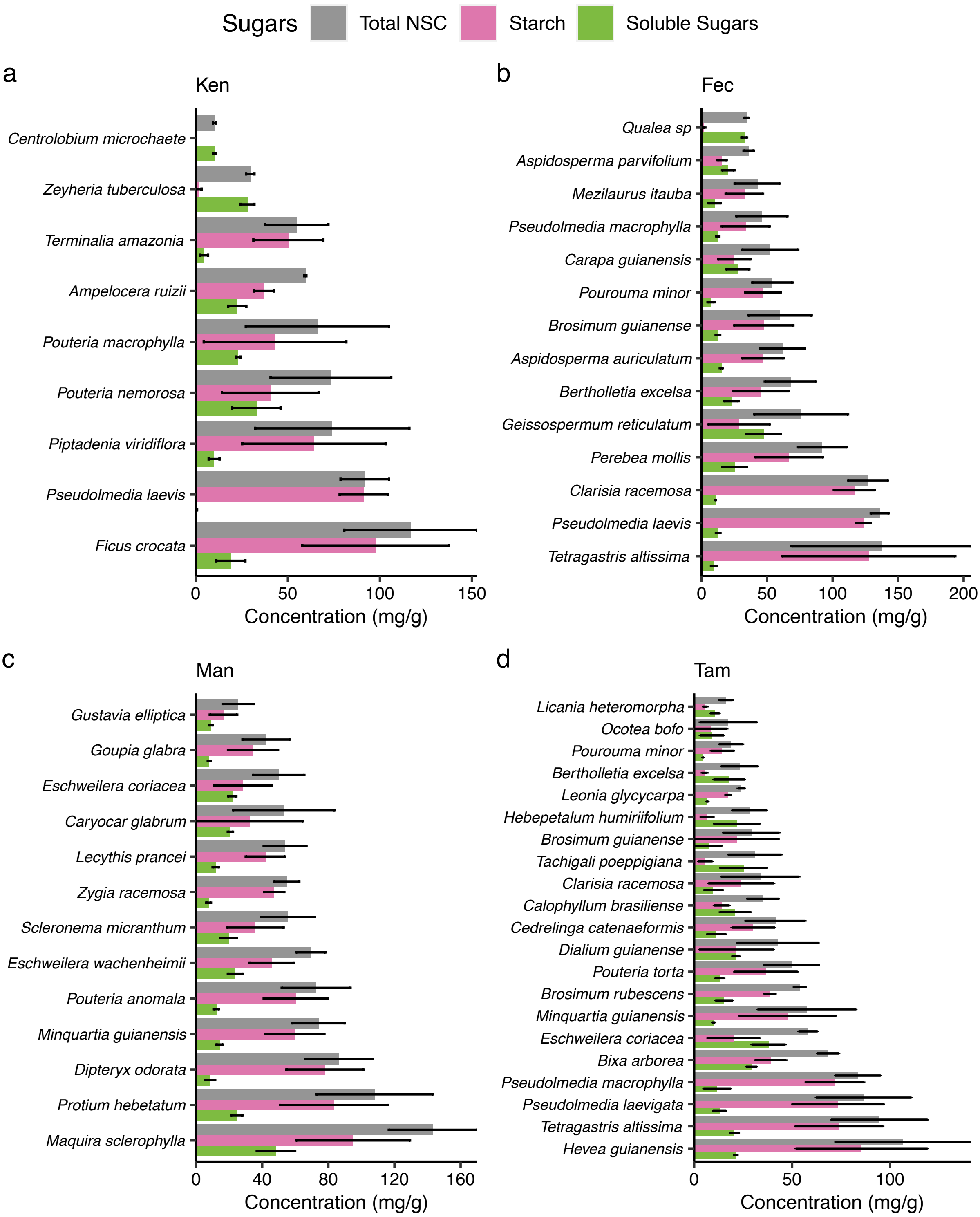

Sugars
Total NSC
Starch
Soluble Sugars
a
Ken
Centrolobium microchaete
Zeyheria tuberculosa
Terminalia amazonia
Ampelocera ruizii
Pouteria macrophylla
Pouteria nemorosa
Piptadenia viridiflora
Pseudolmedia laevis
Ficus crocata
0
50
100
150
Concentration (mg/g)
b
Fec
Qualea sp
Aspidosperma parvifolium
Mezilaurus itauba
Pseudolmedia macrophylla
Carapa guianensis
Pourouma minor
Brosimum guianense
Aspidosperma auriculatum
Bertholletia excelsa
Geissospermum reticulatum
Perebea mollis
Clarisia racemosa
Pseudolmedia laevis
Tetragastris altissima
0
50
100
150
200
Concentration (mg/g)
c
Man
Gustavia elliptica
Goupia glabra
Eschweilera coriacea
Caryocar glabrum
Lecythis prancei
Zygia racemosa
Scleronema micranthum
Eschweilera wachenheimii
Pouteria anomala
Minquartia guianensis
Dipteryx odorata
Protium hebetatum
Maquira sclerophylla
0
40
80
120
160
Concentration (mg/g)
d
Tam
Licania heteromorpha
Ocotea bofo
Pourouma minor
Bertholletia excelsa
Leonia glycycarpa
Hebepetalum humiriifolium
Brosimum guianense
Tachigali poeppigiana
Clarisia racemosa
Calophyllum brasiliense
Cedrelinga catenaeformis
Dialium guianense
Pouteria torta
Brosimum rubescens
Minquartia guianensis
Eschweilera coriacea
Bixa arborea
Pseudolmedia macrophylla
Pseudolmedia laevigata
Tetragastris altissima
Hevea guianensis
0
50
100
Concentration (mg/g)

…. continue

…continuation

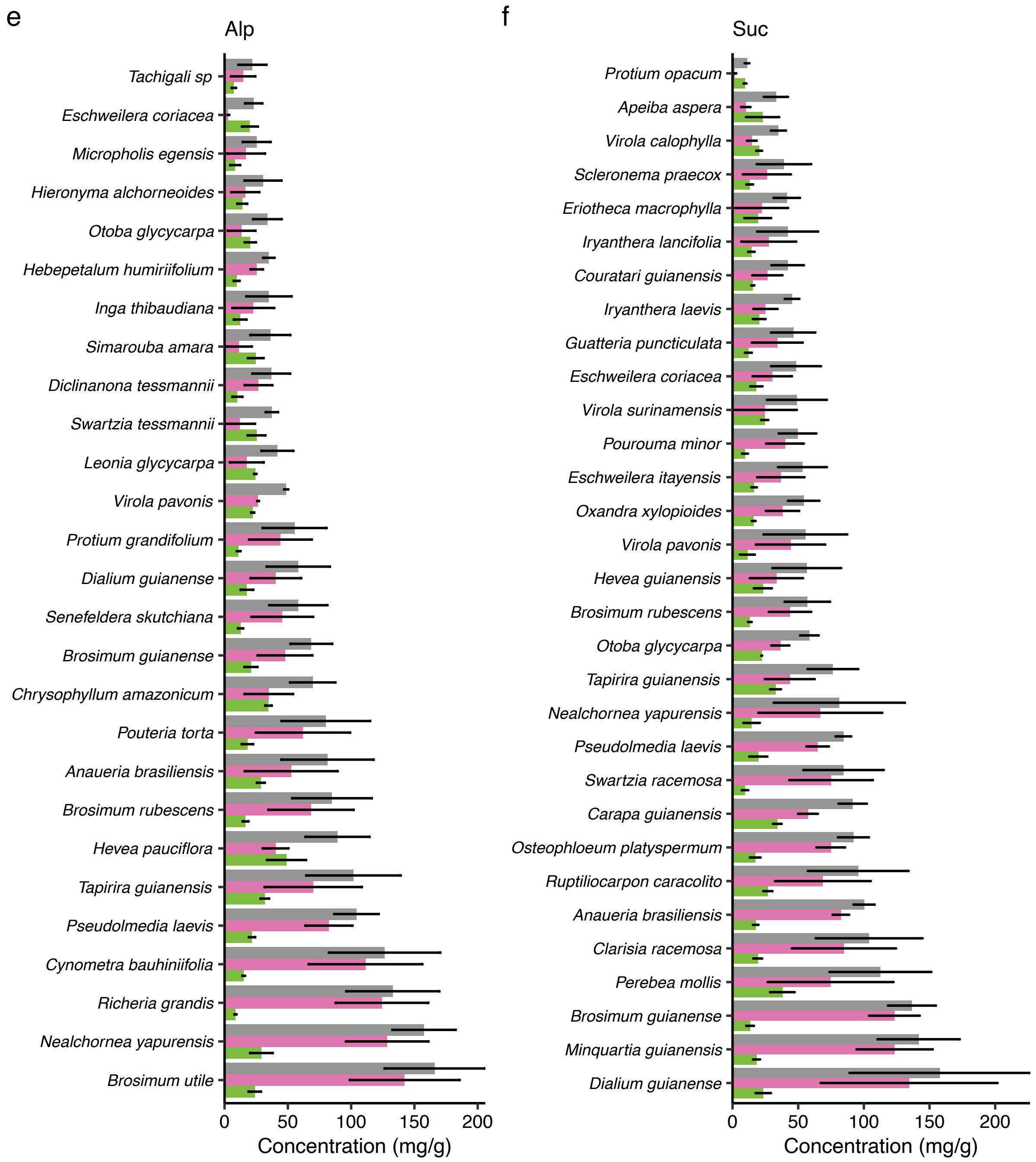

Supplementary Fig. 4 | Wet season mean branch total non-structural carbohydrates (NSC_T), starch and soluble sugar (SS) concentrations in all species of each site. Each panel represent one site, as follow: a) Ken, b) Fec, c) Man, d) Tam, e) Alp and f) Suc. Colours represent NSC_T (grey), starch (pink) and SS (green). Horizontal black bars denotate one standard error of the mean.

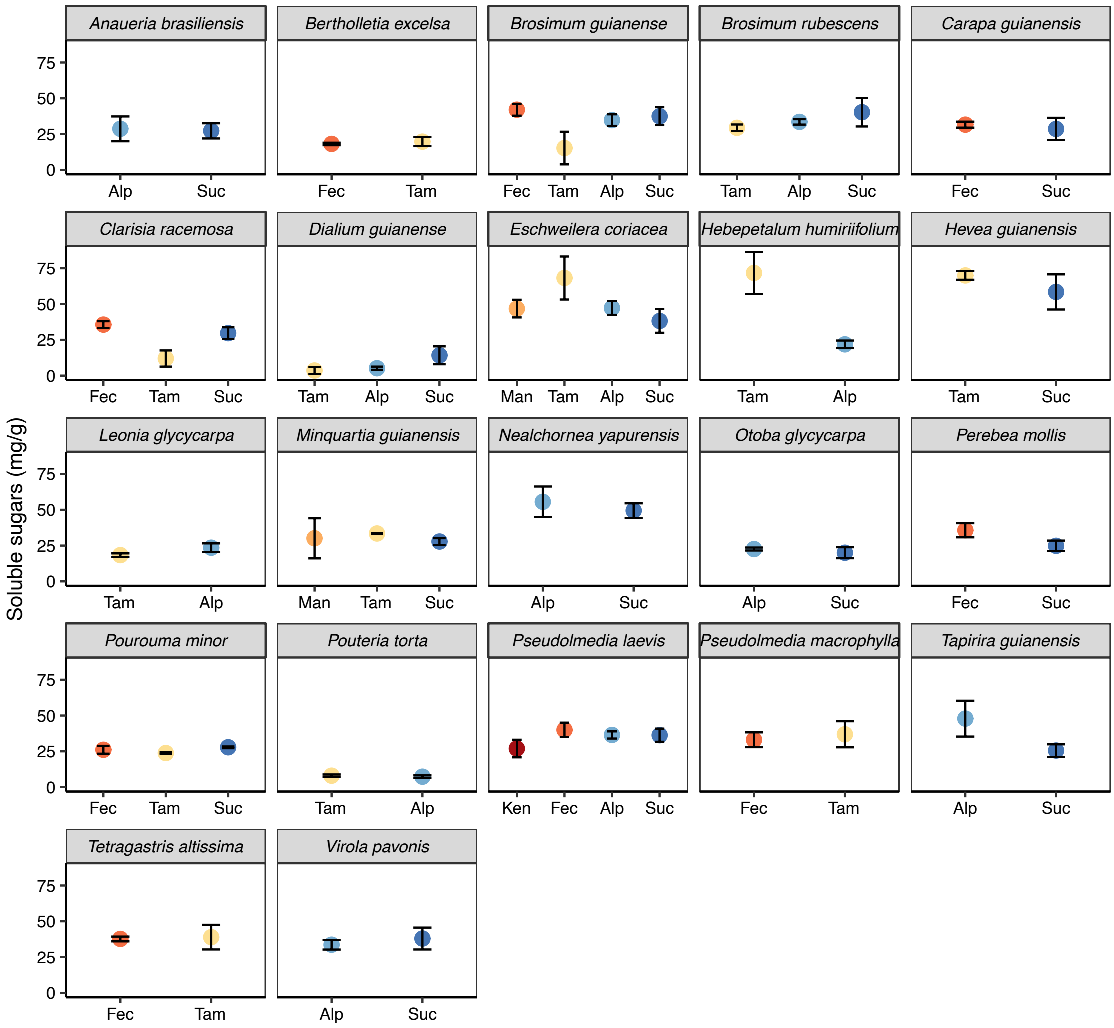

Supplementary Fig. 5 | Comparison of leaf soluble sugars concentrations in species common to more than one site in the wet season. Vertical bars denote one standard error of the mean. We used Kruskal-Wallis test to compare the common species between sites, p-value is displayed for the species that differed between sites and $n \geq 3$.

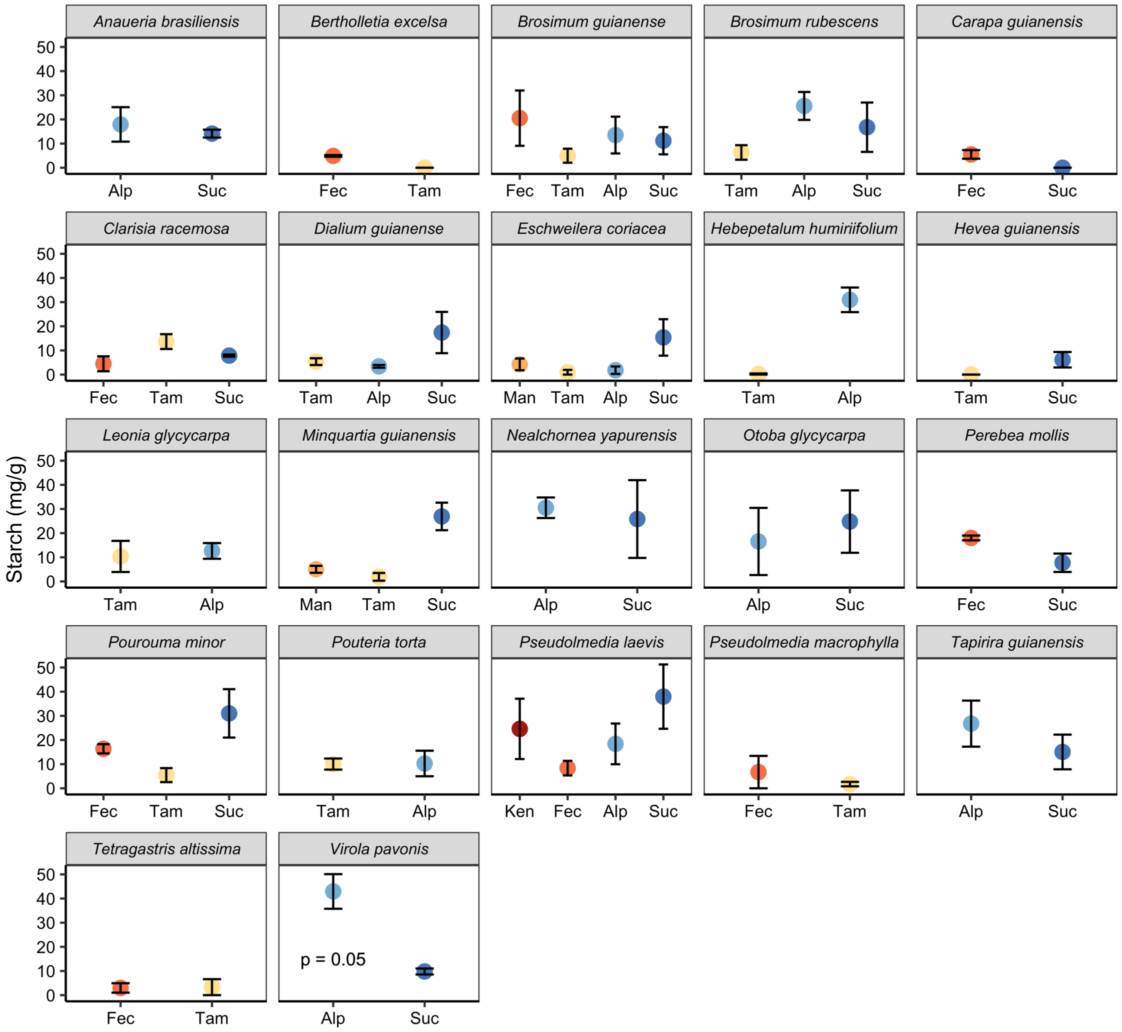

Supplementary Fig. 6 | Comparison of leaf starch concentrations in species common to more than one site in the wet season. Vertical bars denote one standard error of the mean. We used Kruskal-Wallis test to compare the common species between sites, p-value is displayed for the species that differed between sites and $n \geq 3$.

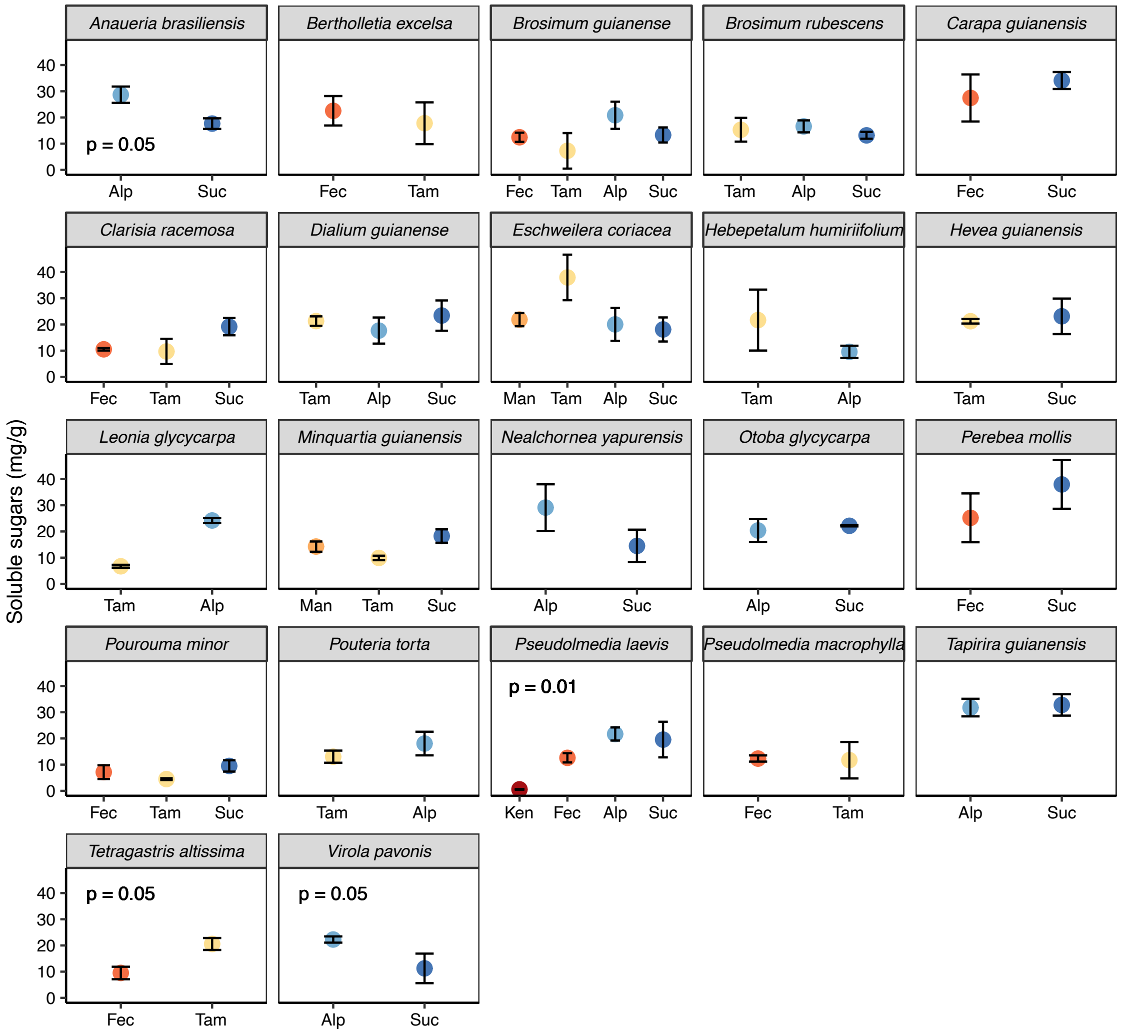

Supplementary Fig. 7 | Comparison of branch soluble sugars concentrations in species common to more than one site in the wet season. Vertical bars denote one standard error of the mean. We used Kruskal-Wallis test to compare the common species between sites, p-value is displayed for the species that differed between sites and $n \geq 3$.

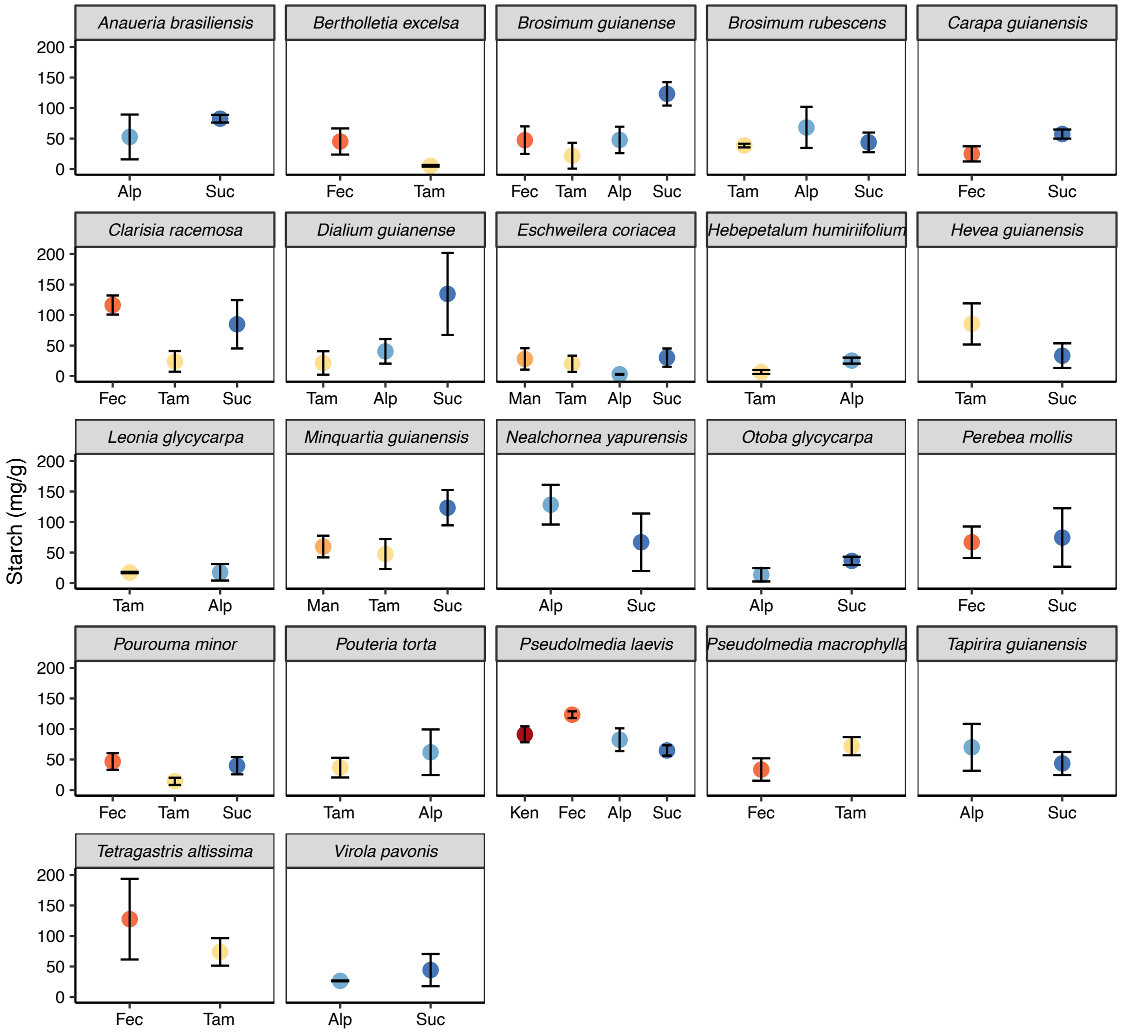

Supplementary Fig. 8 | Comparison of branch starch concentrations in species common to more than one site in the wet season. Vertical bars denote one standard error of the mean. We used Kruskal-Wallis test to compare the common species between sites, p-value is displayed for the species that differed between sites and $n \geq 3$.

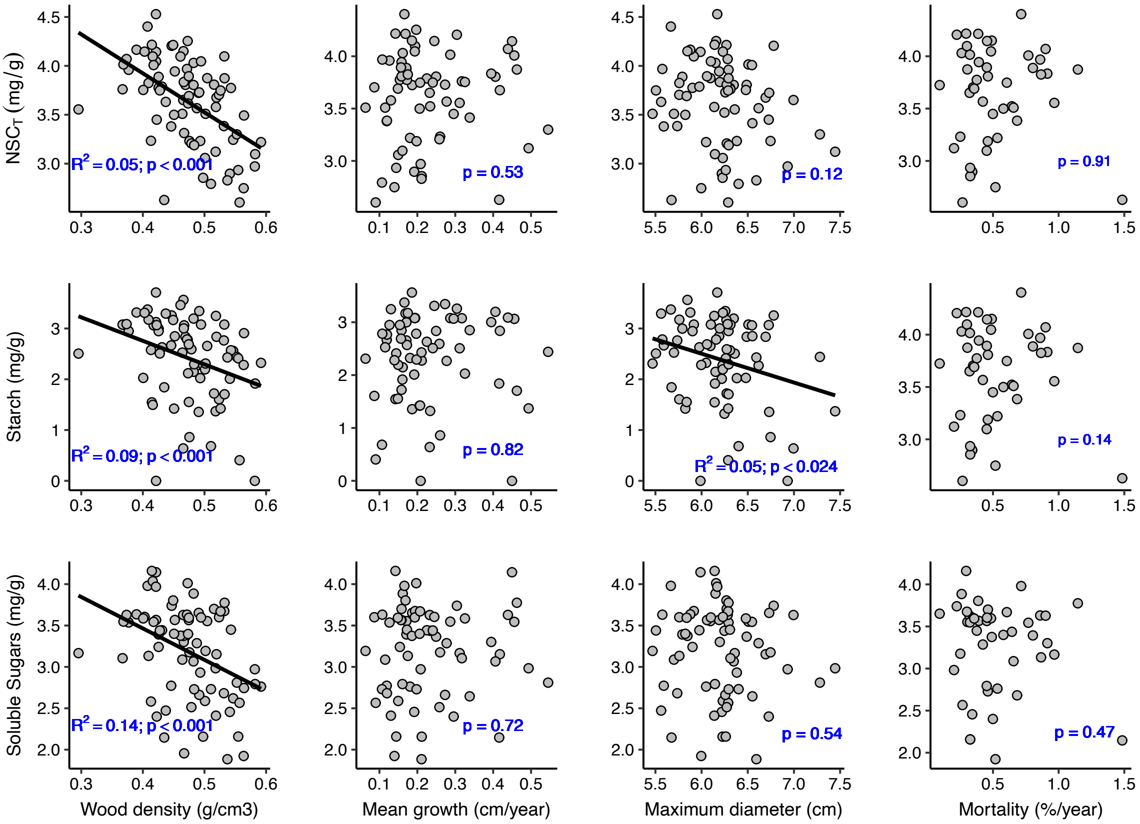

Supplementary Fig. 9 | Bivariate relationships between leaf NSC (total NSC, starch and soluble sugars) and species demographic traits. This figure depicts the NSC relationships with: branch wood density (n= 77 species; data from Tavares et al. in prep. and Barros et al. unpublished data), mean growth rate (n=67 species); potential tree size (n=71 species) and mortality rate (n=45 species) all the last three traits are from Coelho de Souza et al.[3,4]. NSC data used to construct this figure are from wet season. The relationship between NSC and traits was fitted using linear regression, with R^2 displayed only for significant relationships. To construct this figure and to test the relationship we log1p transformed all NSC and traits values. As wet season NSC concentrations did not differ much within individual species across sites, we used the species mean values for these analyses.

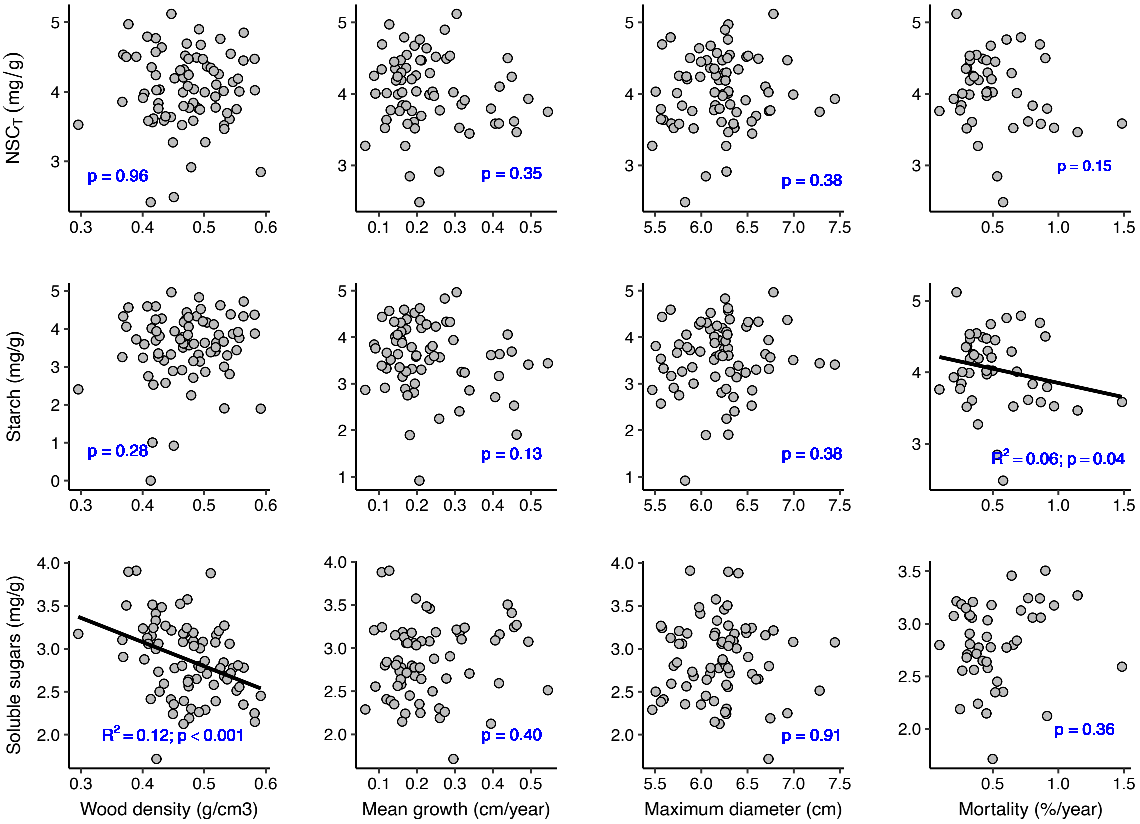

Supplementary Fig. 10 | Bivariate relationships between branch NSC (total NSC, starch and soluble sugars) and species demographic traits. This figure depicts the NSC relationships with: branch wood density (n= 77 species; data from Tavares et al. in prep. and Barros et al. unpublished data), mean growth rate (n=67 species); potential tree size (n=71 species) and mortality rate (n=45 species) all the last three traits are from Coelho de Souza et al.[3,4]. NSC data used to construct this figure are from wet season. The relationship between NSC and traits was fitted using linear regression, with R^2 displayed only for significant relationships. To construct this figure and to test the relationship we log1p transformed all NSC and traits values. As wet season NSC concentrations did not differ much within individual species across sites, we used the species mean values for these analyses.

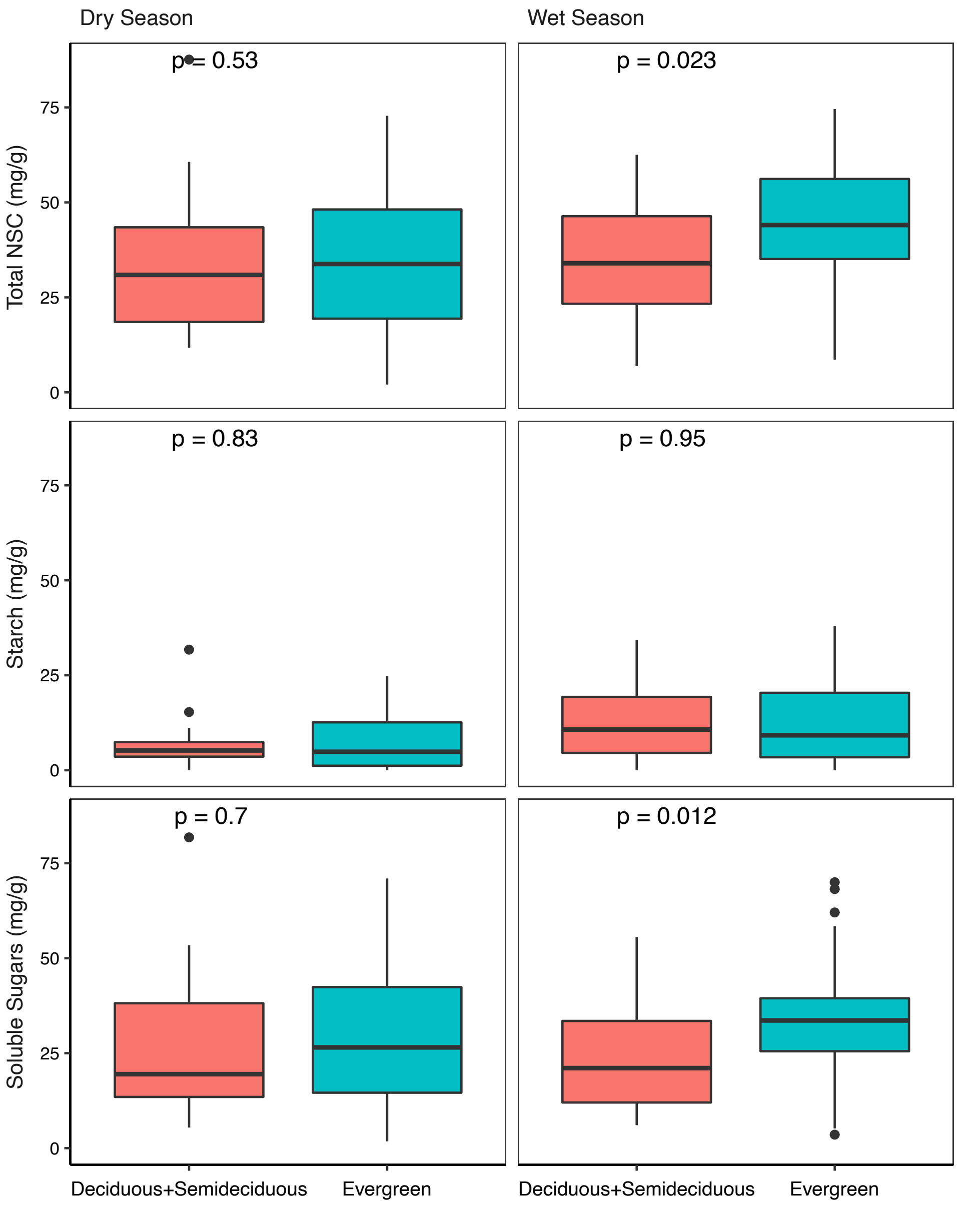

Supplementary Fig. 11 | Comparison of leaf total NSC, starch and soluble sugars between evergreen (n = 25), and Semi-deciduous/deciduous species (n = 15). Comparison between the phenological strategies was calculated using Kruskal-Wallis test. Each box encompasses the 25th to 75th percentiles; the median is indicated by the horizontal line with each box while external horizontal lines indicate the 10th and 90th percentiles; dots indicate outliers.

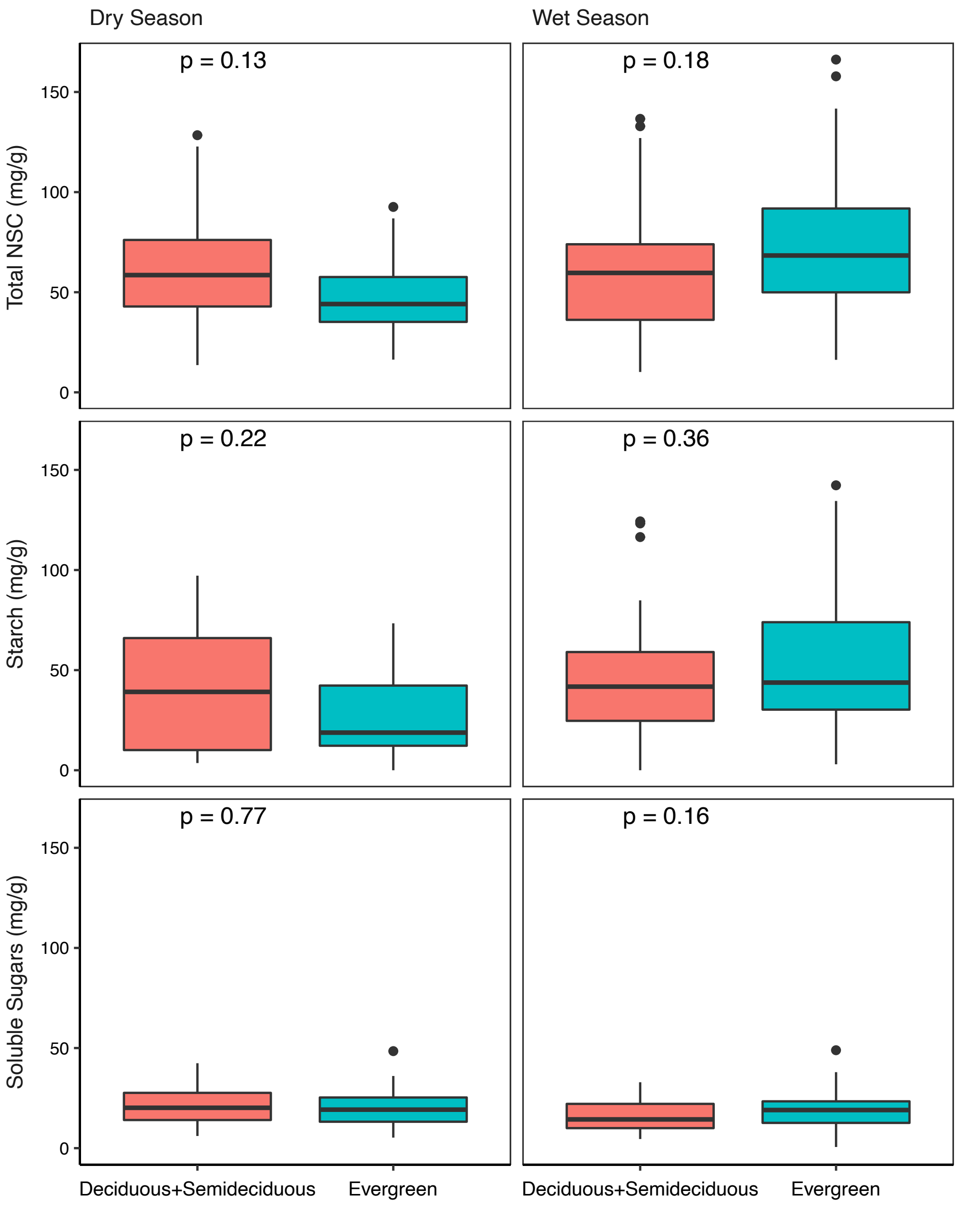

Supplementary Fig. 12 | Comparison of branch total NSC, starch and soluble sugars between evergreen (n = 25), and Semi-deciduous/deciduous species (n = 15). Comparison between the phenological strategies was calculated using Kruskal-Wallis test. Each box encompasses the 25th to 75th percentiles; the median is indicated by the horizontal line with each box while external horizontal lines indicate the 10th and 90th percentiles; dots indicate outliers.

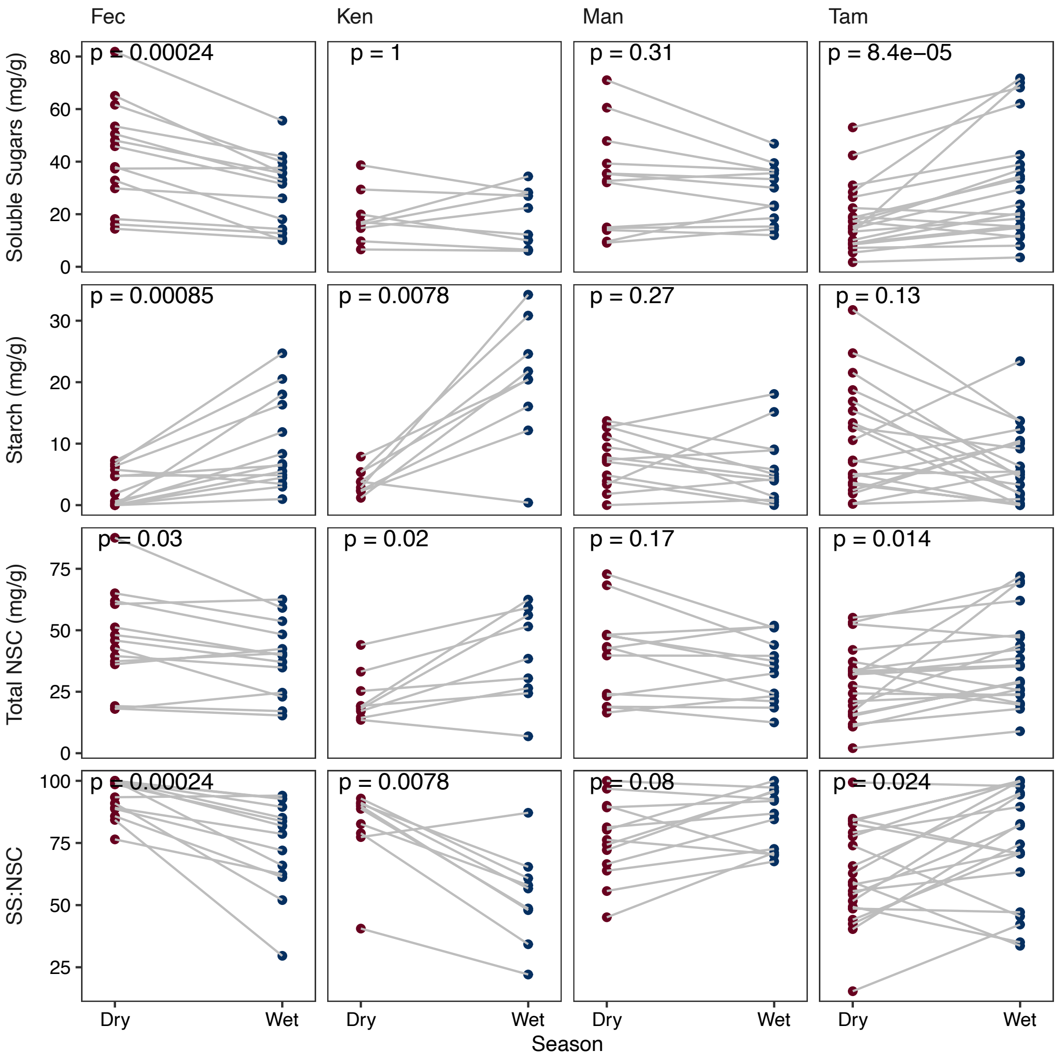

Supplementary Fig. 13 | Leaf total NSC, starch, soluble sugars, and SS:NSC$_T$ change from wet to dry season across species in the Ken, Fec, Man and Tam site. Lines connect NSC fraction values for individual species between seasons. Each dot represents one species. Values of significance for seasonal comparison are shown for each site x NSC component (paired samples Wilcoxon test).

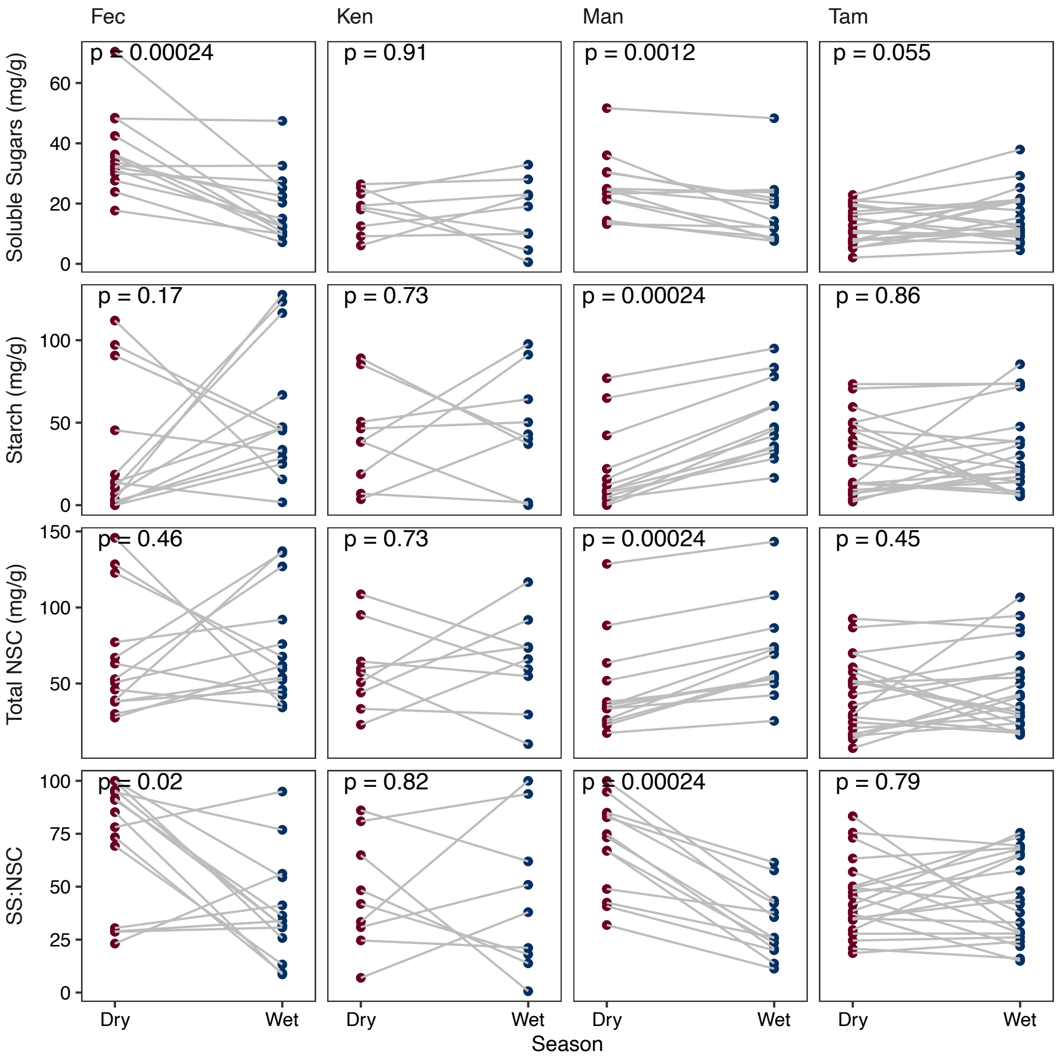

Supplementary Fig. 14 | Branch total NSC, starch, soluble sugars (SS) and SS:NSC$_T$ change from wet to dry season across species in the Ken, Fec, Man and Tam site. Lines connect NSC fraction values for individual species between seasons. Each dot represents on species. Values of significance for seasonal comparison are shown for each site x NSC component (paired samples Wilcoxon test).

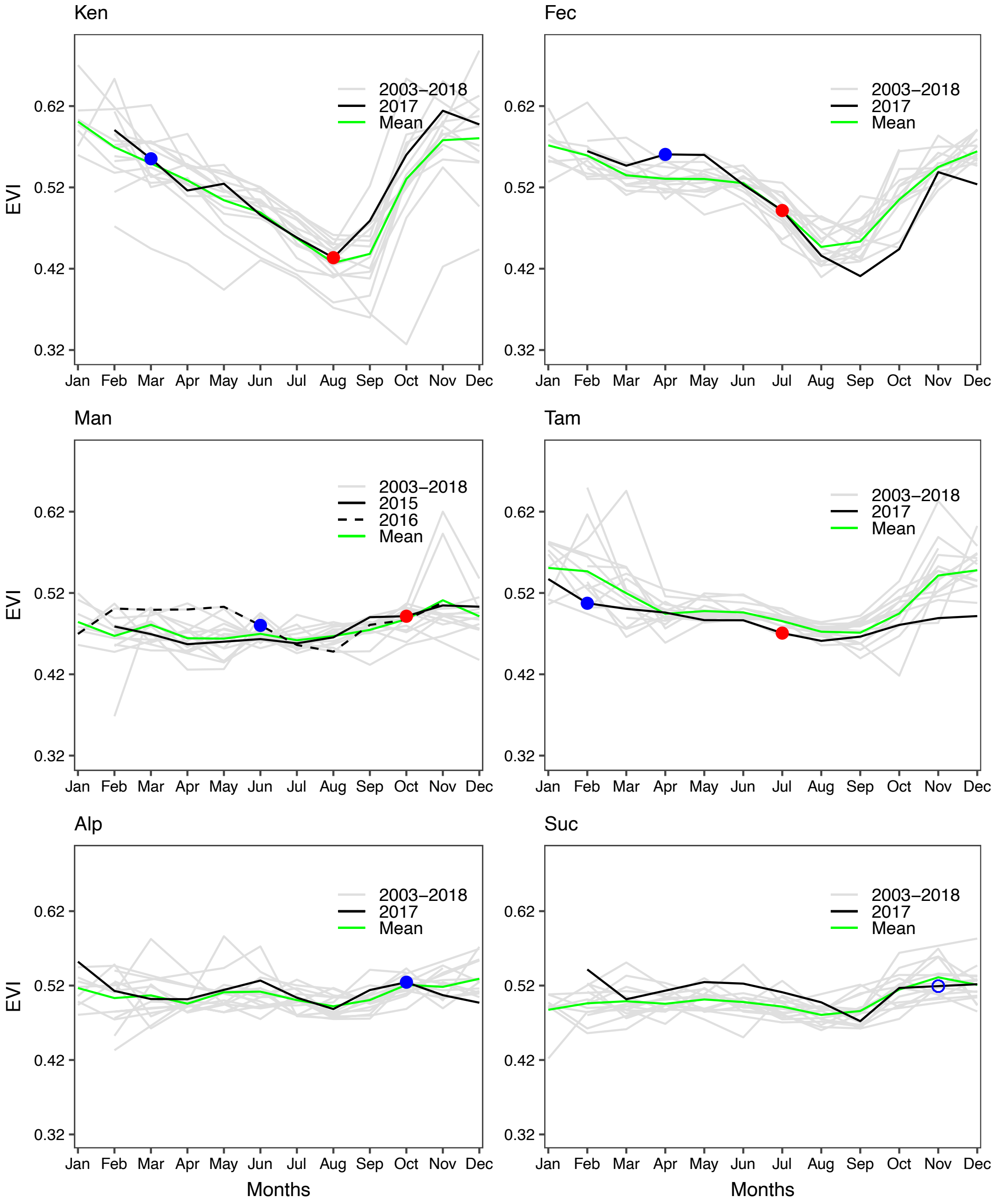

Supplementary Fig. 15 | Enhanced vegetation index for our study sites (EVI). Grey lines represent EVI from 2003 to 2018. The green line represents the mean EVI for all years and black line is the mean EVI for the year in which sample collection took place. Blue and red dots denote the sampling month for the wet and dry season respectively. For the SUC site, EVI data were unavailable for the sampling month, and this site is thus represented by an open circle. To construct these figures we used the data available in Dalagnol et al.[5,6].

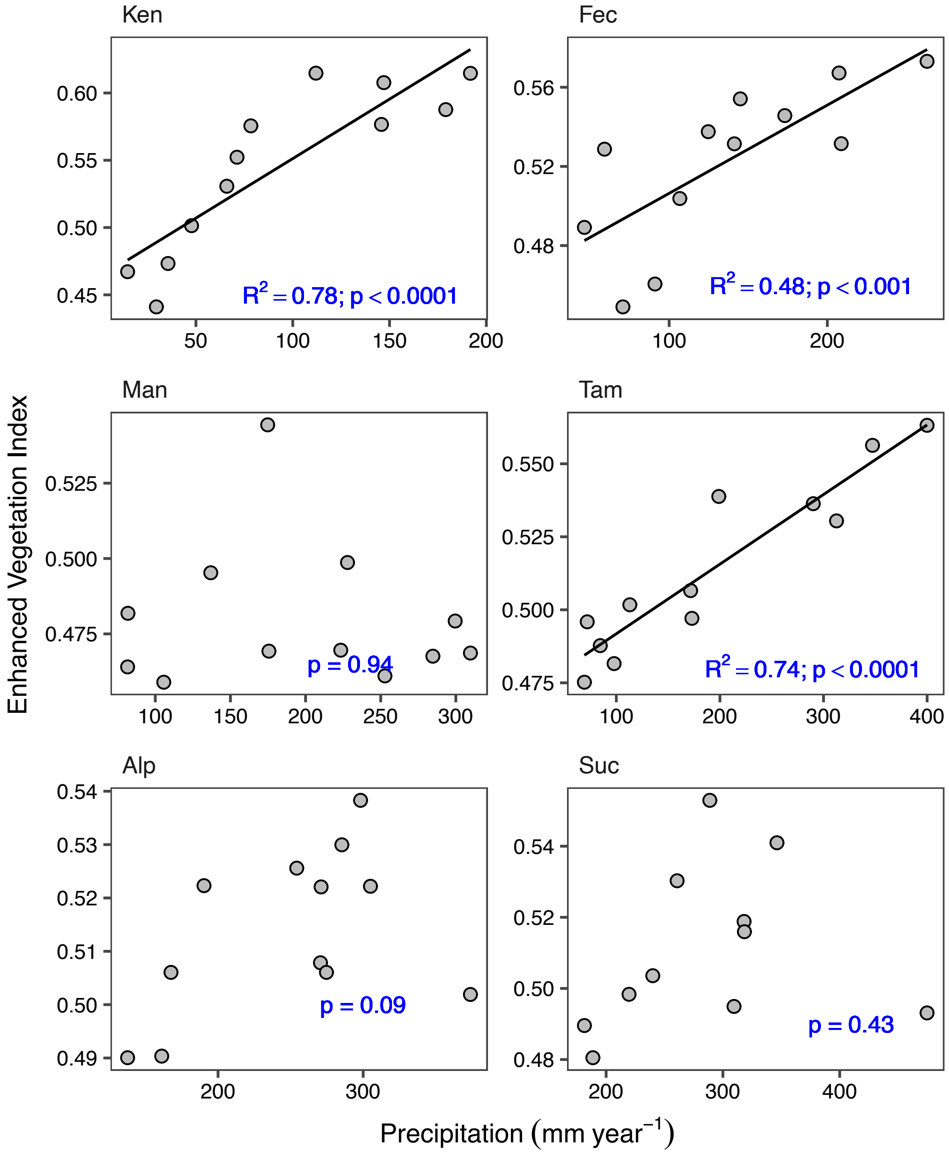

Supplementary Fig. 16 | Relationship between monthly enhanced vegetation index (EVI) and precipitation (mm/month). The relationship between EVI and precipitation was fitted using linear models. EVI and precipitation data are from 2013 to 2018. EVI data is from in Dalagnol et al.[5,6] and precipitation data from CRU-TS 4.03[1] downscaled with WorldClim 2.1[2].

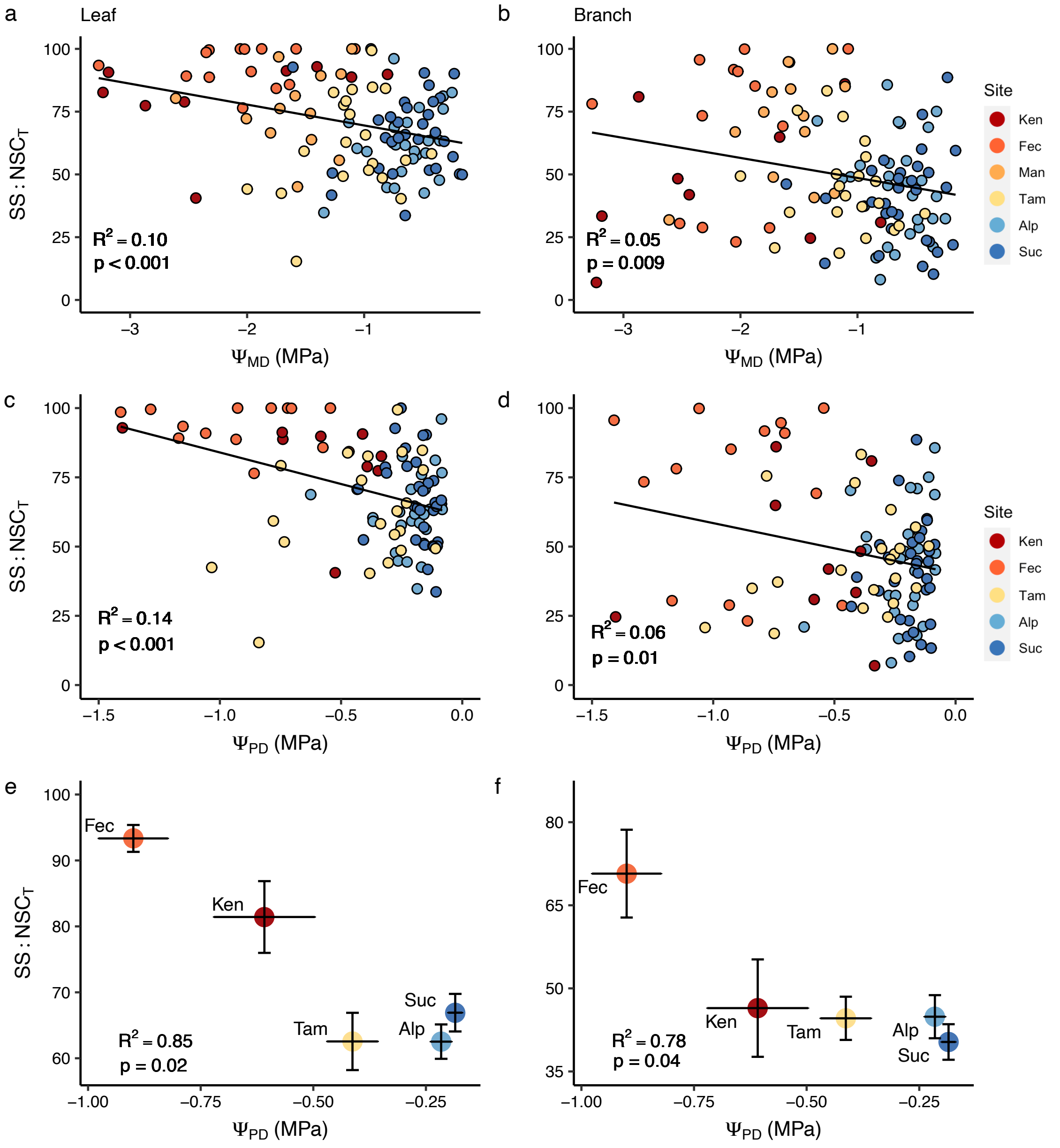

Supplementary Fig. 17 | Relationship in leaves (left) and branches (right) between proportion of NSC$_T$ corresponding to soluble sugars (SS:NSC$_T$) and leaf water potential (Ψ). Panels a-b) SS:NSC$_T$ relationship with midday leaf water potential (Ψ_{MD}) at species level; c-d) SS:NSC$_T$ relationship with pre-dawn leaf water potential (Ψ_{PD}) at species level; e-f) SS:NSC$_T$ relationship with Ψ_{PD} at community-level. Vertical bars denote one standard error of the mean. The relationship between SS:NSC$_T$ and Ψ was fitted with standardized major axis (SMA) regression. Ψ_{PD} was not measured in MAN.

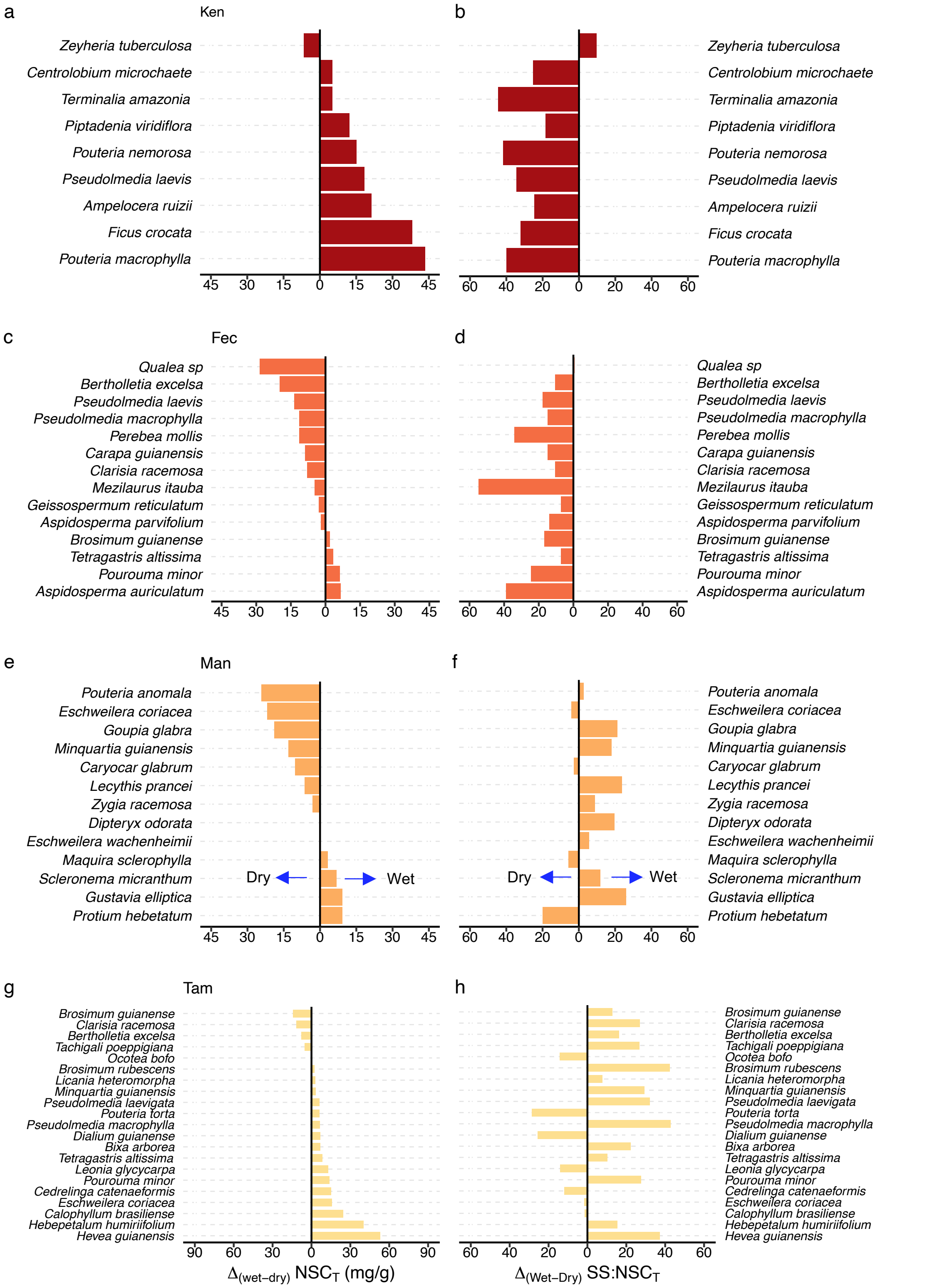

Supplementary Fig. 18 | Seasonal trends in allocation of total NSC (NSC_T) and proportion of NSC_T in the form of soluble sugars ($SS:NSC_T$) in leaves. From top to bottom sites are ordered from driest to wettest. To construct these figures, we used the difference between wet and dry seasons. Bars to the left of zero indicate increased NSC_T and $SS:NSC_T$ during the dry season while bars to the right of zero indicate increased in the wet season.

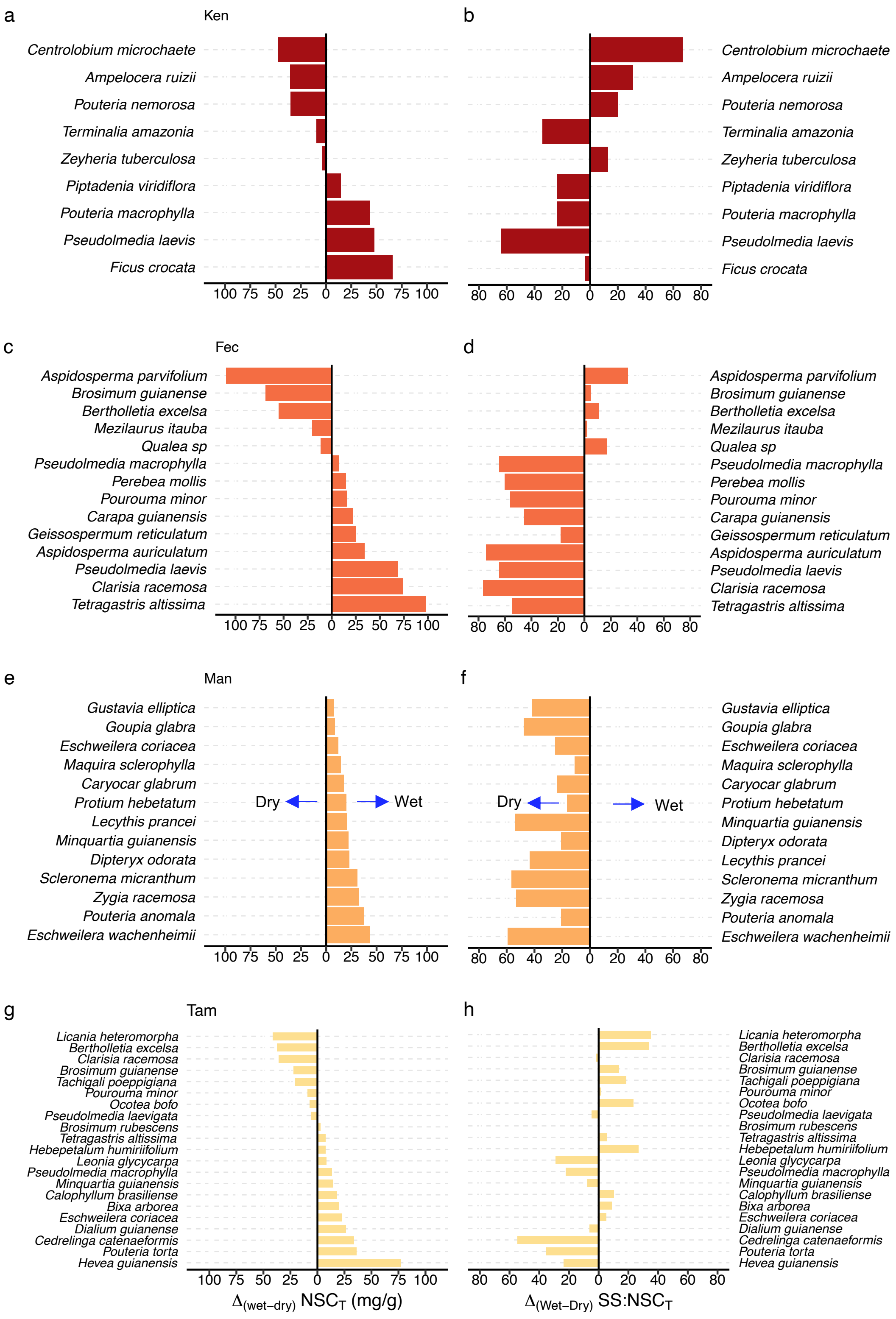

Supplementary Fig. 19 | Seasonal trends in allocation of total NSC (NSC_T) and proportion of NSC_T in the form of soluble sugars ($SS:NSC_T$) in branches. From top to bottom sites are ordered from driest to wettest. To construct these figures, we used the difference between wet and dry seasons. Bars to the left of zero indicate increased NSC_T and %Soluble Sugars during the dry season while bars to the right of zero indicate increased in the wet season.

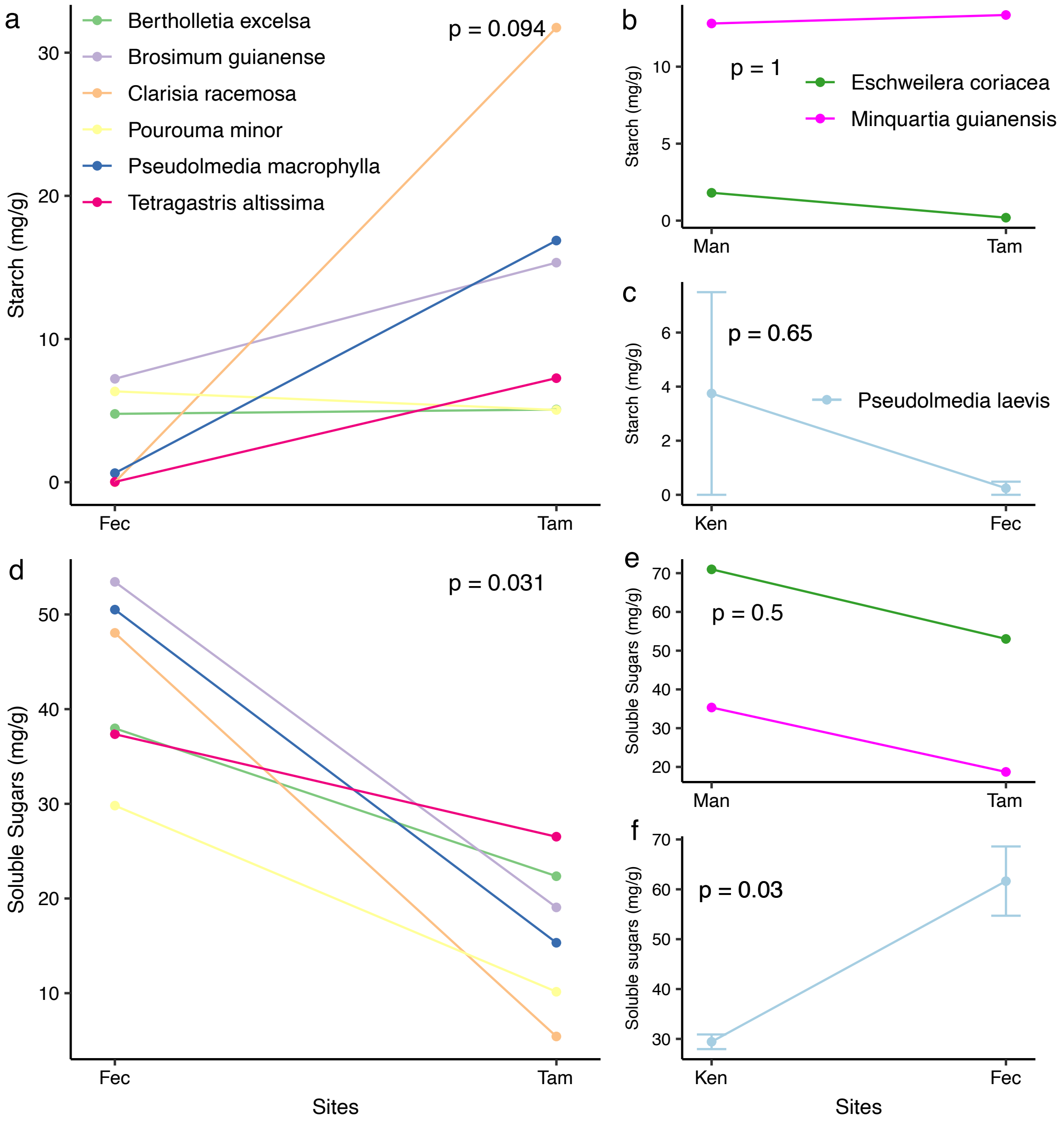

Supplementary Fig. 20 | Differences in leaf starch and soluble sugars concentrations in species common to more than one site in the dry season. Panels a-c represent the starch concentrations and panels d-f soluble sugar concentrations. Each colour represents one species. Paired samples Wilcoxon test was used for comparison between sites. Vertical lines in the panels c and f represent one standard error of the mean.

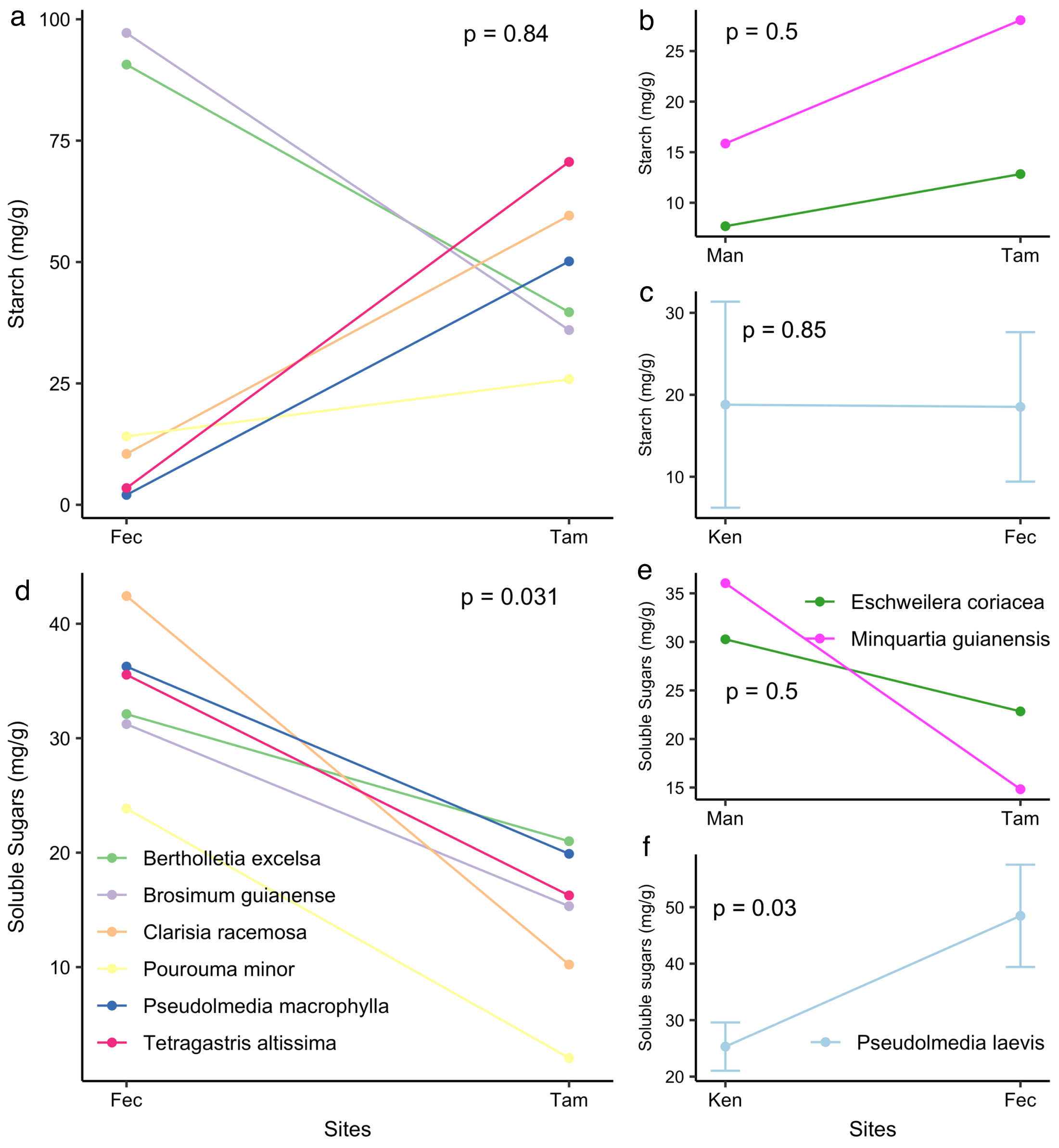

Supplementary Fig. 21 | Differences in branch starch and soluble sugars concentrations in species common to more than one site in the dry season. Panels a-c represent the starch concentrations and panels d-f soluble sugar concentrations. Each colour represents one species. Paired samples Wilcoxon test was used for comparison between sites. Vertical lines in the panels c and f represent one standard error of the mean.

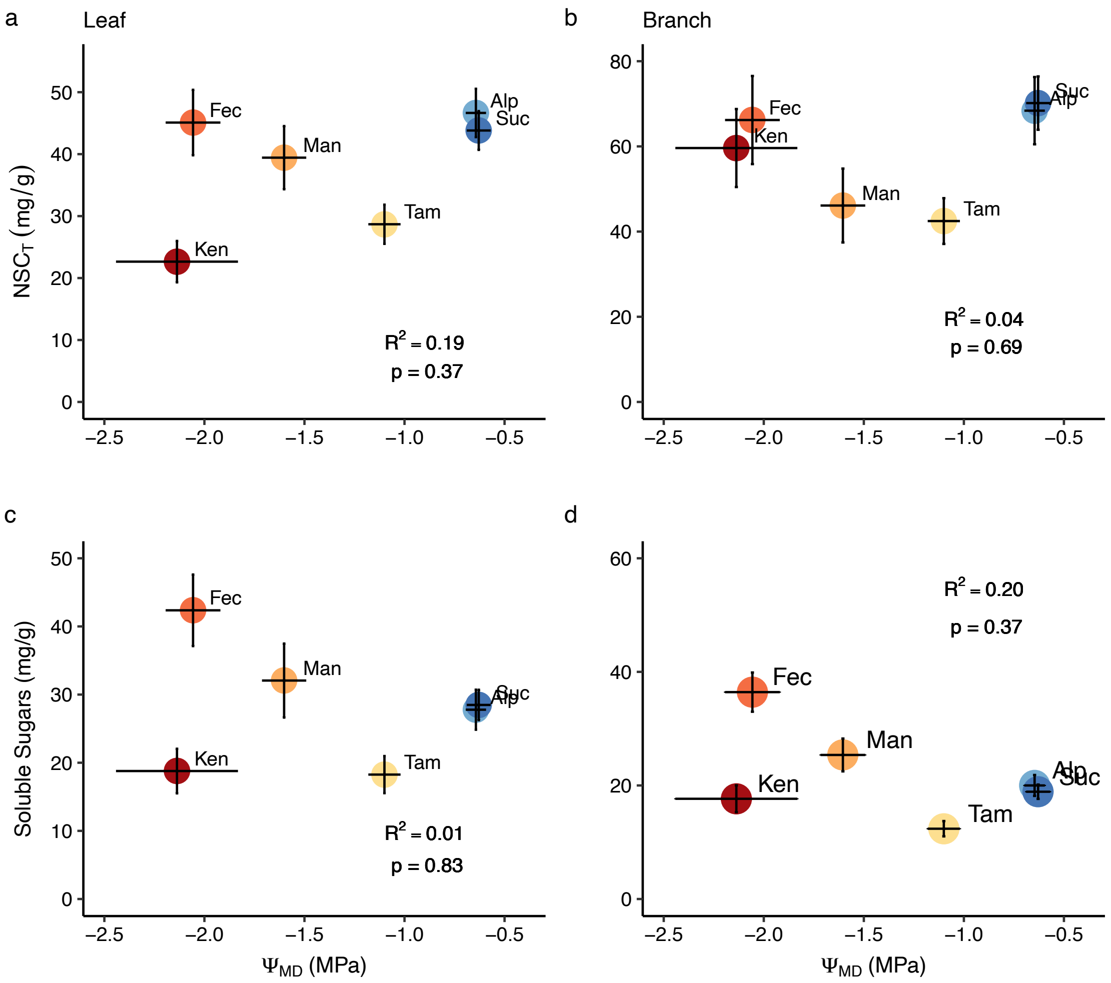

Supplementary Fig. 22 | Relationship between NSC and midday leaf water potential (Ψ_{MD}). Relationship between Ψ_{MD} a) leaf total NSC (NSC_T) and b) branch NSC_T; c) leaf soluble sugar (SS) and d) branch SS concentrations. The NSC_T, SS and Ψ_{MD} represent the mean of all species sampled in each site in the dry season, except in the two sites where there is no dry season (monthly precipitation < 100 mm month^{-1}). The relationship between NSC_T and SS with Ψ_{MD} was fitted with standardized major axis (SMA). Vertical and horizontal bars denote one standard error of the mean.

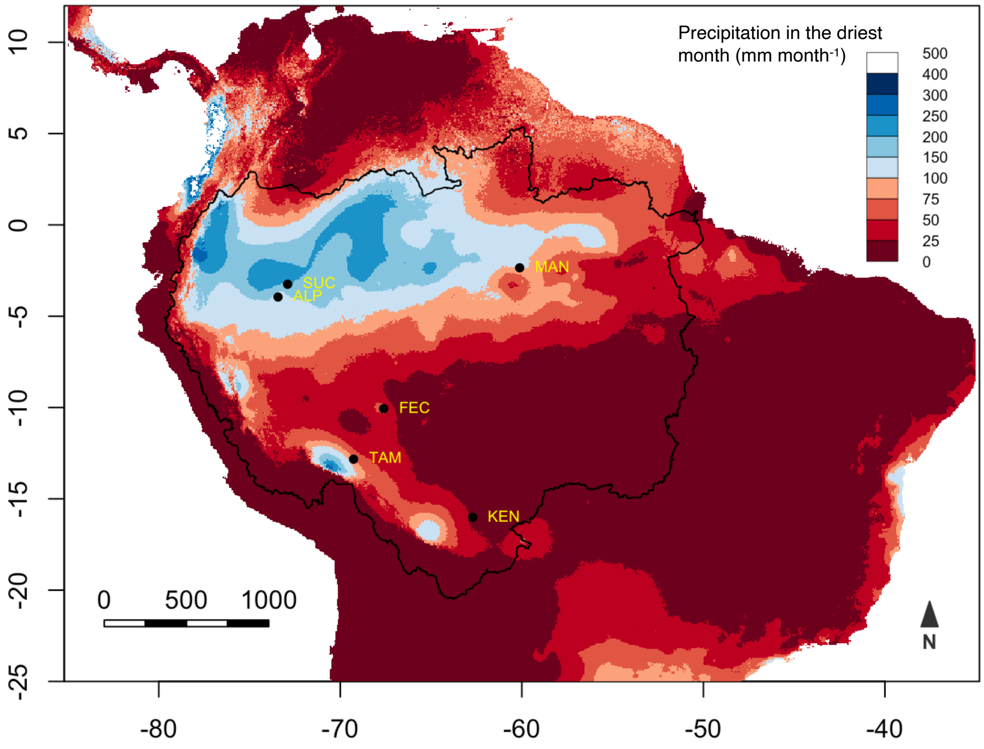

Supplementary Fig. 23 | Location of sampled sites and precipitation of driest month. The map depicts precipitation of driest month in South America (mm month^{-1}). The Amazon basin is bounded by the black outline. Data for the map is derived from WorldClim v2 (1970-2010, 30s resolution)[2].

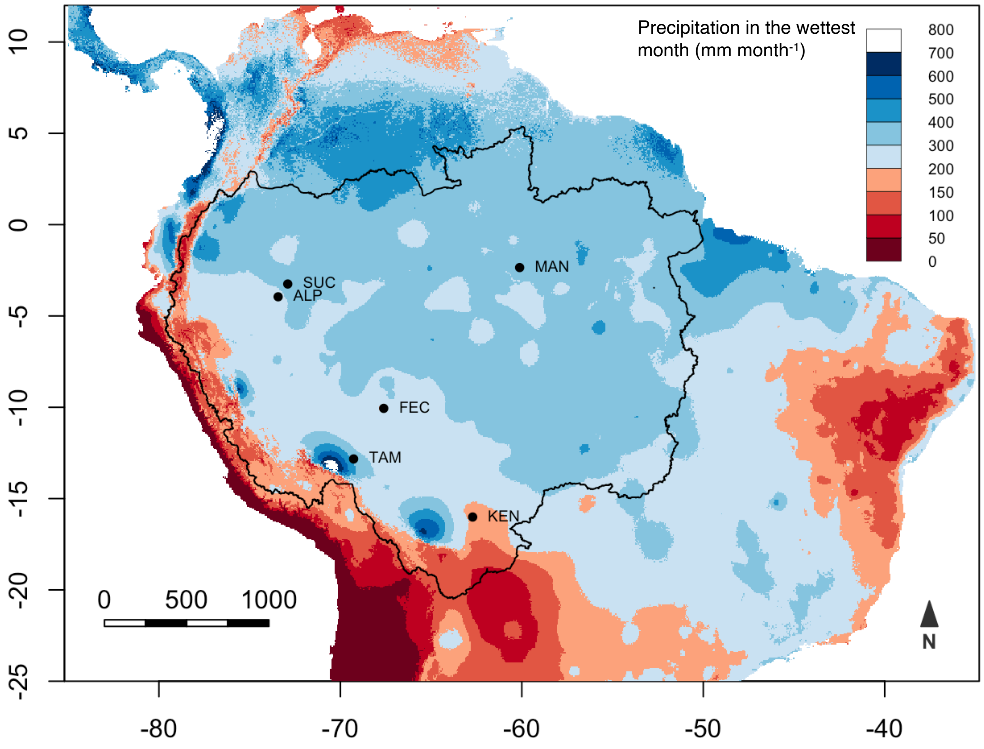

Supplementary Fig. 24 | Location of sampled sites and precipitation of wettest month. The map depicts precipitation of wettest month in South America (mm month $^{-1}$). The Amazon basin is bounded by the black outline. Data for the map is derived from WorldClim v2 (1970-2010, 30s resolution)[2].

SUPPLEMENTARY TABLES

Supplementary Table 1 | Site, species names, family, phenology and number of trees sampled (*n*).

Site	Species	Family	Phenology	*n*
Alp	*Anaueria brasiliensis*	Lauraceae	Unknown	3
Alp	*Brosimum guianense*	Moraceae	Deciduous	3
Alp	*Brosimum rubescens*	Moraceae	Evergreen	3
Alp	*Brosimum utile*	Moraceae	Evergreen	3
Alp	*Chrysophyllum amazonicum*	Sapotaceae	Unknown	3
Alp	*Cynometra bauhiniifolia*	Fabaceae	Unknown	3
Alp	*Dialium guianense*	Fabaceae	Evergreen	3
Alp	*Diclinanona tessmannii*	Annonaceae	Unknown	3
Alp	*Eschweilera coriacea*	Lecythidaceae	Evergreen	3
Alp	*Hebepetalum humiriifolium*	Linaceae	Unknown	6
Alp	*Hevea pauciflora*	Euphorbiaceae	Evergreen	3
Alp	*Hieronyma alchorneoides*	Phyllanthaceae	Evergreen	3
Alp	*Inga thibaudiana*	Fabaceae	Unknown	3
Alp	*Leonia glycycarpa*	Violaceae	Unknown	3
Alp	*Micropholis egensis*	Sapotaceae	Unknown	2
Alp	*Nealchornea yapurensis*	Euphorbiaceae	Unknown	3
Alp	*Otoba glycycarpa*	Myristicaceae	Unknown	3
Alp	*Pouteria torta*	Sapotaceae	Deciduous	3
Alp	*Protium grandifolium*	Burseraceae	Evergreen	3
Alp	*Pseudolmedia laevis*	Moraceae	Evergreen	6
Alp	*Richeria grandis*	Phyllanthaceae	Semi-deciduous	3
Alp	*Senefeldera skutchiana*	Euphorbiaceae	Unknown	3
Alp	*Simarouba amara*	Simaroubaceae	Evergreen	3
Alp	*Swartzia tessmannii*	Fabaceae	Unknown	2
Alp	*Tachigali ind.*	Fabaceae	Unknown	3
Alp	*Tapirira guianensis*	Anacardiaceae	Evergreen	3
Alp	*Virola pavonis*	Myristicaceae	Unknown	3
Fec	*Aspidosperma auriculatum*	Apocynaceae	Unknown	2
Fec	*Aspidosperma parvifolium*	Apocynaceae	Unknown	3
Fec	*Bertholletia excelsa*	Lecythidaceae	Deciduous	3
Fec	*Brosimum guianense*	Moraceae	Deciduous	3
Fec	*Carapa guianensis*	Meliaceae	Evergreen	2
Fec	*Clarisia racemosa*	Moraceae	Semi-deciduous	3
Fec	*Geissospermum reticulatum*	Apocynaceae	Unknown	3
Fec	*Mezilaurus itauba*	Lauraceae	Evergreen	3
Fec	*Perebea mollis*	Moraceae	Unknown	3

Fec	*Pourouma minor*	Urticaceae	Unknown	3
Fec	*Pseudolmedia laevis*	Moraceae	Evergreen	4
Fec	*Pseudolmedia macrophylla*	Moraceae	Unknown	2
Fec	*Qualea sp*	Vochysiaceae	Deciduous	3
Fec	*Tetragastris altissima*	Burseraceae	Evergreen	3
Ken	*Ampelocera ruizii*	Ulmaceae	Semi-deciduous	2
Ken	*Centrolobium microchaete*	Fabaceae	Deciduous	2
Ken	*Ficus crocata*	Moraceae	Evergreen	3
Ken	*Piptadenia viridiflora*	Fabaceae	Deciduous	3
Ken	*Pouteria macrophylla*	Sapotaceae	Semi-deciduous	3
Ken	*Pouteria nemorosa*	Sapotaceae	Semi-deciduous	3
Ken	*Pseudolmedia laevis*	Moraceae	Evergreen	3
Ken	*Terminalia amazonia*	Combretaceae	Semi-deciduous	3
Ken	*Zeyheria tuberculosa*	Bignoniaceae	Semi-deciduous	3
Man	*Caryocar glabrum*	Caryocaraceae.	Unknown	2
Man	*Dipteryx odorata*	Fabaceae	Evergreen	2
Man	*Eschweilera coriacea*	Lecythidaceae	Evergreen	3
Man	*Eschweilera wachenheimii*	Lecythidaceae	Unknown	2
Man	*Goupia glabra*	Goupiaceae	Semi-deciduous	3
Man	*Gustavia elliptica*	Lecythidaceae	Unknown	3
Man	*Lecythis prancei*	Lecythidaceae	Evergreen	2
Man	*Maquira sclerophylla*	Moraceae	Unknown	3
Man	*Minquartia guianensis*	Olacaceae	Evergreen	2
Man	*Pouteria anomala*	Sapotaceae	Evergreen	3
Man	*Protium hebetatum*	Burseraceae	Unknown	3
Man	*Scleronema micranthum*	Bombacaceae	Unknown	3
Man	*Zygia racemosa*	Fabaceae	Unknown	2
Suc	*Anaueria brasiliensis*	Lauraceae	Unknown	3
Suc	*Apeiba aspera*	Malvaceae	Unknown	3
Suc	*Brosimum guianense*	Moraceae	Deciduous	3
Suc	*Brosimum rubescens*	Moraceae	Evergreen	3
Suc	*Carapa guianensis*	Meliaceae	Evergreen	2
Suc	*Clarisia racemosa*	Moraceae	Semi-deciduous	3
Suc	*Couratari guianensis*	Lecythidaceae	Deciduous	3
Suc	*Dialium guianense*	Fabaceae	Evergreen	3
Suc	*Eriotheca macrophylla*	Malvaceae	Unknown	2
Suc	*Eschweilera coriacea*	Lecythidaceae	Evergreen	6
Suc	*Eschweilera itayensis*	Lecythidaceae	Unknown	3
Suc	*Guatteria puncticulata*	Annonaceae	Evergreen	3
Suc	*Hevea guianensis*	Euphorbiaceae	Evergreen	3
Suc	*Iryanthera laevis*	Myristicaceae	Unknown	3
Suc	*Iryanthera lancifolia*	Myristicaceae	Unknown	2

Suc	*Minquartia guianensis*	Olacaceae	Evergreen	3
Suc	*Nealchornea yapurensis*	Euphorbiaceae	Unknown	3
Suc	*Osteophloeum platyspermum*	Myristicaceae	Unknown	3
Suc	*Otoba glycycarpa*	Myristicaceae	Unknown	3
Suc	*Oxandra xylopioides*	Annonaceae	Unknown	3
Suc	*Perebea mollis*	Moraceae	Unknown	3
Suc	*Pourouma minor*	Urticaceae	Unknown	3
Suc	*Protium opacum*	Burseraceae	Unknown	3
Suc	*Pseudolmedia laevis*	Moraceae	Evergreen	3
Suc	*Ruptiliocarpon caracolito*	Lepidobotryaceae	Unknown	3
Suc	*Scleronema praecox*	Malvaceae	Unknown	3
Suc	*Swartzia racemosa*	Fabaceae	Unknown	3
Suc	*Tapirira guianensis*	Anacardiaceae	Evergreen	3
Suc	*Virola calophylla*	Myristicaceae	Unknown	3
Suc	*Virola pavonis*	Myristicaceae	Unknown	3
Suc	*Virola surinamensis*	Myristicaceae	Evergreen	3
Tam	*Bertholletia excelsa*	Lecythidaceae	Deciduous	2
Tam	*Bixa arborea*	Urticaceae	Evergreen	3
Tam	*Brosimum guianense*	Moraceae	Deciduous	2
Tam	*Brosimum rubescens*	Moraceae	Evergreen	3
Tam	*Calophyllum brasiliense*	Clusiaceae	Evergreen	3
Tam	*Cedrelinga catenaeformis*	Meliaceae	Unknown	3
Tam	*Clarisia racemosa*	Moraceae	Semi-deciduous	3
Tam	*Dialium guianense*	Fabaceae	Evergreen	3
Tam	*Eschweilera coriacea*	Lecythidaceae	Evergreen	3
Tam	*Hebepetalum humiriifolium*	Linaceae	Unknown	3
Tam	*Hevea guianensis*	Euphorbiaceae	Evergreen	2
Tam	*Leonia glycycarpa*	Violaceae	Unknown	3
Tam	*Licania heteromorpha*	Chrysobalanaceae	Evergreen	3
Tam	*Minquartia guianensis*	Olacaceae	Evergreen	3
Tam	*Ocotea bofo*	Lauraceae	Unknown	2
Tam	*Pourouma minor*	Urticaceae	Unknown	2
Tam	*Pouteria torta*	Sapotaceae	Deciduous	3
Tam	*Pseudolmedia laevigata*	Moraceae	Evergreen	3
Tam	*Pseudolmedia macrophylla*	Moraceae	Unknown	3
Tam	*Tachigali poeppigiana*	Fabaceae	Unknown	3
Tam	*Tetragastris altissima*	Burseraceae	Evergreen	3

Supplementary Table 2 | Comparison of total NSC (NSC_T), starch, soluble sugars (SS) and proportion of NSC_T in the form of SS ($SS:NSC_T$) among sites. Differences between sites were calculated for each organ and season using the Kruskal-Wallis test at 95% confidence level.

Organ	Season	Sugar	*Chi²*	*df*	*p-value*
Leaf	Dry	NSC_T	19.491	5	**0.001**
		Starch	44.285	5	**<0.001**
		SS	21.155	5	**< 0.001**
		SS: NSC_T	39.157	5	**< 0.001**
	Wet	NSC_T	0.923	3	0.8198
		Starch	11.913	3	0.0077
		SS	3.540	3	0.3156
		SS: NSC_T	11.106	3	**0.011**
Branch	Dry	NSC_T	10.512	5	0.0620
		Starch	14.906	5	**0.010**
		SS	40.839	5	**< 0.001**
		SS: NSC_T	18.760	5	**0.002**
	Wet	NSC_T	7.441	3	0.0591
		Starch	5.854	3	0.1189
		SS	0.712	3	0.8705
		SS: NSC_T	1.958	3	0.5813

Supplementary Table 3 | Comparison of total NSC (NSC_T), starch, soluble sugars (SS) and SS:NSC_T (proportion of NSC_T in the form of SS) among species within site. Differences among species were calculated for each organ and season using the Kruskal-Wallis test at 95% confidence level.

Organ	Site	Season	Sugar	*Chi²*	*df*	*p-value*
Leaf	Ken	Dry	NSC_T	16.01	8	**0.042**
			Starch	5.00	8	0.757
			SS	18.17	8	**0.019**
			SS:NSC_T	6.53	8	0.587
		Wet	NSC_T	20.38	8	**0.008**
			Starch	10.38	8	0.238
			SS	19.14	8	**0.014**
			SS:NSC_T	12.11	8	0.146
	Fec	Dry	NSC_T	33.47	13	**0.001**
			Starch	25.19	13	**0.021**
			SS	34.34	13	**0.001**
			SS:NSC_T	25.92	13	**0.017**
		Wet	NSC_T	33.74	13	**0.001**
			Starch	25.58	13	**0.019**
			SS	33.29	13	**0.001**
			SS:NSC_T	28.43	13	**0.007**
	Man	Dry	NSC_T	24.34	12	**0.018**
			Starch	16.87	12	0.154
			SS	26.43	12	**0.009**
			SS:NSC_T	21.79	12	**0.039**
		Wet	NSC_T	17.76	12	0.122
			Starch	15.52	12	0.213
			SS	18.78	12	0.093
			SS:NSC_T	18.83	12	0.092
	Tam	Dry	NSC_T	35.79	20	**0.016**
			Starch	36.17	20	**0.014**
			SS	34.62	20	**0.022**
			SS:NSC_T	26.32	20	0.155
		Wet	NSC_T	40.92	20	**0.003**
			Starch	34.57	20	**0.022**
			SS	42.81	20	**0.002**
			SS:NSC_T	37.82	20	**0.009**
	Alp	Wet	NSC_T	54.01	26	**0.001**
			Starch	33.45	26	0.149
			SS	71.00	26	**<0.001**
			SS:NSC_T	31.47	26	0.211
	Suc	Wet	NSC_T	53.47	30	**0.005**
			Starch	36.97	30	0.177
			SS	64.16	30	**<0.001**
			SS:NSC_T	46.73	30	**0.026**

Branch	Ken	Dry	NSC_T	7.95	8	0.437
			Starch	11.24	8	0.188
			SS	12.15	8	0.144
			$SS:NSC_T$	13.78	8	0.087
		Wet	NSC_T	11.28	8	0.185
			Starch	14.00	8	0.081
			SS	17.62	8	**0.024**
			$SS:NSC_T$	18.95	8	**0.015**
	Fec	Dry	NSC_T	30.19	13	**0.004**
			Starch	28.67	13	**0.007**
			SS	26.01	13	**0.016**
			$SS:NSC_T$	28.37	13	**0.008**
		Wet	NSC_T	18.35	13	0.144
			Starch	21.88	13	0.057
			SS	27.31	13	**0.011**
			$SS:NSC_T$	25.17	13	**0.021**
	Man	Dry	NSC_T	21.48	12	**0.043**
			Starch	15.63	12	0.208
			SS	19.36	12	0.080
			$SS:NSC_T$	17.13	12	0.144
		Wet	NSC_T	17.84	12	0.120
			Starch	12.75	12	0.387
			SS	28.21	12	**0.005**
			$SS:NSC_T$	13.29	12	0.347
	Tam	Dry	NSC_T	27.20	20	0.129
			Starch	25.20	20	0.193
			SS	30.61	20	**0.060**
			$SS:NSC_T$	22.69	20	0.303
		Wet	NSC_T	33.35	20	**0.030**
			Starch	30.35	20	0.064
			SS	28.10	20	0.106
			$SS:NSC_T$	27.31	20	0.126
	Alp	Wet	NSC_T	47.24	26	**0.006**
			Starch	39.96	26	**0.039**
			SS	56.67	26	**<0.001**
			$SS:NSC_T$	38.04	26	0.060
	Suc	Wet	NSC_T	40.53	30	0.094
			Starch	37.88	30	0.152
			SS	46.78	30	**0.026**
			$SS:NSC_T$	40.92	30	0.088

Supplementary Table 4 | Number (*n*) of species with deciduous, evergreen, semi-deciduous or unknown phenology in each site. Phenological data were available for 40 out 84 tree species sampled. Of these, 25 species were classed as 'evergreen', eight as 'semi-deciduous' and seven as 'deciduous' species, distribute across sites as shown in the table below.

Site	Phenology	*n*
Ken	Deciduous	2
	Evergreen	2
	Semi-deciduous	5
Fec	Deciduous	3
	Evergreen	4
	Semi-deciduous	1
	Unknown	6
Man	Evergreen	5
	Semi-deciduous	1
	Unknown	7
Tam	Deciduous	3
	Evergreen	10
	Semi-deciduous	1
	Unknown	7
Alp	Deciduous	2
	Evergreen	10
	Semi-deciduous	1
	Unknown	14
Suc	Deciduous	2
	Evergreen	10
	Semi-deciduous	1
	Unknown	18

Supplementary Table 5 | Total NSC (NSC_T), starch and soluble sugars (SS) concentrations and proportion of NSC_T in the form of SS ($SS:NSC_T$) and seasonal comparison within site. *P* denotes the significance for paired samples Wilcoxon test at 95% confidence level. Bold values represent statistically differences in mean NSC between dry and wet season.

Organ	Sugar	Season	KEN	*p*	*v*	FEC	*p*	*v*	MAN	*p*	*v*	TAM	*p*	*v*	ALP	SUC
Leaf	NSC_T	Dry	22.6 ± 3.3	**0.019**	3	45.1 ± 5.3	**0.029**	87	39.4 ± 5.1	0.167	66	28.7 ± 3.2	**0.014**	46	-	-
		Wet	39.5 ± 6.3			38.5 ± 3.9			34.0 ± 3.7			37.5 ± 4.0			46.14 ± 3.82	43.72 ± 3.15
	Starch	Dry	3.8 ± 0.6	**0.007**	1	2.7 ± 0.8	**<0.001**	4	7.4 ± 1.2	0.273	62	10.4 ± 1.2	0.128	160	-	-
		Wet	20.1 ± 3.3			9.7 ± 1.2			6.0 ± 1.5			6.7 ± 1.3			18.43 ± 1.82	15.32 ± 1.76
	SS	Dry	18.8 ± 3.3	1.000	23	42.4 ± 5.2	**<0.001**	104	32.0 ± 5.4	0.305	61	18.2 ± 2.7	**<0.001**	13	-	-
		Wet	19.4 ± 3.6			28.8 ± 3.7			28.0 ± 3.1			31.0 ± 4.6			27.71 ± 2.90	28.40 ± 2.24
	SS: NSC_T	Dry	81.4 ± 5.4	**0.007**	44	93.3 ± 2.0	**<0.001**	104	76.3 ± 4.4	0.080	20	62.5 ± 4.3	**0.023**	51	-	-
		Wet	53.4 ± 6.2			74.4 ± 4.9			84.3 ± 3.4			74.6 ± 5.0			62.91 ± 2.59	66.80 ± 2.85
Branch	NSC_T	Dry	59.6 ± 9.2	0.734	19	66.2 ± 10.3	0.463	40	46.1 ± 8.7	**<0.001**	0	42.5 ± 5.4	0.452	93	-	-
		Wet	64.0± 10.5			73.0± 9.7			68.6 ± 8.5			47.6 ± 5.9			68.15 ± 7.90	70.41 ± 6.26
	Starch	Dry	42.0 ± 10.2	0.734	19	29.7 ± 10.7	0.172	30	20.7 ± 7.9	**<0.001**	0	30.1 ± 4.9	0.864	110	-	-
		Wet	47.2 ± 11.3			54.1 ± 10.8			50.7 ± 6.5			31.5 ± 5.3			48.23 ± 7.52	51.50 ± 5.99
	SS	Dry	17.6 ± 2.4	0.910	21	36.4 ± 3.4	**<0.001**	104	25.4 ± 2.9	**0.001**	88	12.4 ± 1.3	0.054	60	-	-
		Wet	16.8 ± 3.7			18.9 ± 3.0			17.7 ± 3.1			16.1 ± 1.8			19.92 ± 1.82	18.90 ± 1.26
	SS: NSC_T	Dry	46.4 ± 8.8	0.820	25	70.7 ± 7.9	**0.020**	89	68.7 ± 6.0	**<0.001**	91	44.6 ± 3.9	0.785	107	-	-
		Wet	44.2 ± 11.8			38.9 ± 6.6			32.2 ± 4.4			44.5 ± 4.5			44.73 ± 3.83	40.10 ± 3.25

1. Harris, I., Jones, P. D., Osborn, T. J. & Lister, D. H. Updated high-resolution grids of monthly climatic observations - the CRU TS3.10 Dataset. *Int. J. Climatol.* (2014). doi:10.1002/joc.3711
2. Fick, S. E. & Hijmans, R. J. WorldClim 2: new 1-km spatial resolution climate surfaces for global land areas. *Int. J. Climatol.* **37**, 4302–4315 (2017).
3. Coelho de Souza, F. *et al.* Evolutionary heritage influences Amazon tree ecology. *Proc. R. Soc. B Biol. Sci.* **283**, 20161587 (2016).
4. Coelho de Souza, F. *et al.* Trait data from: 'Evolutionary heritage influences Amazon tree ecology'. *ForestPlots.net* (2016). doi:10.5521/FORESTPLOTS.NET/2016_4
5. Dalagnol, R., Wagner, F. H., Galvão, L. S. & Aragão, L. E. O. C. The MANVI product: MODIS (MAIAC) nadir-solar adjusted vegetation indices (EVI and NDVI) for South America. *Zenodo* (2019). doi:10.5281/ZENODO.3159488
6. Dalagnol, R., Wagner, F. H., Galvão, L. S., Nelson, B. W. & De Aragão, L. E. O. E. C. Life cycle of bamboo in the southwestern Amazon and its relation to fire events. *Biogeosciences* **15**, 6087–6104 (2018).
