# Supplementary material for: Non-structural carbohydrates mediate seasonal water stress across Amazon forests
Source: Nat Commun. 2021 Apr 19;12:2310. doi: 10.1038/s41467-021-22378-8 (PMC8055652; doi:10.1038/s41467-021-22378-8)
Supplement: Supplementary file 3 — Reporting Summary [file 41467_2021_22378_MOESM3_ESM.pdf]

nature research

Corresponding author(s): Caroline Signori-Müller

Last updated by author(s): 2021/02/18

Reporting Summary

Nature Research wishes to improve the reproducibility of the work that we publish. This form provides structure for consistency and transparency in reporting. For further information on Nature Research policies, see our Editorial Policies and the Editorial Policy Checklist.

Statistics

For all statistical analyses, confirm that the following items are present in the figure legend, table legend, main text, or Methods section.

n/a	Confirmed	
☐	☒	The exact sample size (*n*) for each experimental group/condition, given as a discrete number and unit of measurement
☐	☒	A statement on whether measurements were taken from distinct samples or whether the same sample was measured repeatedly
☐	☒	The statistical test(s) used AND whether they are one- or two-sided<br>*Only common tests should be described solely by name; describe more complex techniques in the Methods section.*
☐	☒	A description of all covariates tested
☐	☒	A description of any assumptions or corrections, such as tests of normality and adjustment for multiple comparisons
☐	☒	A full description of the statistical parameters including central tendency (e.g. means) or other basic estimates (e.g. regression coefficient) AND variation (e.g. standard deviation) or associated estimates of uncertainty (e.g. confidence intervals)
☐	☒	For null hypothesis testing, the test statistic (e.g. *F*, *t*, *r*) with confidence intervals, effect sizes, degrees of freedom and *P* value noted<br>*Give P values as exact values whenever suitable.*
☒	☐	For Bayesian analysis, information on the choice of priors and Markov chain Monte Carlo settings
☒	☐	For hierarchical and complex designs, identification of the appropriate level for tests and full reporting of outcomes
☒	☐	Estimates of effect sizes (e.g. Cohen's *d*, Pearson's *r*), indicating how they were calculated

Our web collection on statistics for biologists contains articles on many of the points above.

Software and code

Policy information about availability of computer code

Data collection	No software was used for data collection.
Data analysis	All data was analysed through open source packages in R software (3.6.0).<br>MODIS and Climatic data pixel values were extracted considering the coordinate system of each site using raster (3.0.12)and rgdal (1.4.8).<br>Post-hoc Mann-Whitney-Wilcoxon Rank Sum test using Bonferroni correction from the Agricolae package(1.3.2) was used to evaluate differences among sites. We plotted figures using ggplot2 (3.3.2) and ggpubr (0.2.5). To calculate the standardized major axis (SMA) regression we used the smatr (3.4.8). The parameters for the variance partition were estimated by the Residual or Restricted Maximum Likelihood (REML) method with the lme4 (1.1.21).

For manuscripts utilizing custom algorithms or software that are central to the research but not yet described in published literature, software must be made available to editors and reviewers. We strongly encourage code deposition in a community repository (e.g. GitHub). See the Nature Research guidelines for submitting code & software for further information.

Data

Policy information about availability of data

All manuscripts must include a data availability statement. This statement should provide the following information, where applicable:

- Accession codes, unique identifiers, or web links for publicly available datasets
- A list of figures that have associated raw data
- A description of any restrictions on data availability

The data of non-structural carbohydrates concentrations are available at www.forestplots.net/data-packages/Signori-Muller-et-al-2021 (DOI 10.5521/forestplots.net/2021_3). The mean growth rate, potential tree size and mortality rate are available at www.forestplots.net/data-packages/coelho-de-souza-et-al-2016 (DOI 10.5521/FORESTPLOTS.NET/2016_4) . The climatic data are available at www.worldclim.org/data/index.html. Enhance vegetation index data are

available on www.zenodo.org/record/3159488#.YBW_u3f7Tlw (DOI 10.5281/ZENODO.3159488). Leaf water potential data are available upon reasonable request to the correspondence author. Branches wood density are from Tavares et al. (in prep). The inventory data for species selection are from the RAINFOR network available upon request at www.forestplots.net.

Field-specific reporting

Please select the one below that is the best fit for your research. If you are not sure, read the appropriate sections before making your selection.

☐ Life sciences ☐ Behavioural & social sciences ☒ Ecological, evolutionary & environmental sciences

For a reference copy of the document with all sections, see nature.com/documents/nr-reporting-summary-flat.pdf

Ecological, evolutionary & environmental sciences study design

All studies must disclose on these points even when the disclosure is negative.

Study description	For the present study we measure leaf water potential and analyse the concentrations of non-structural carbohydrates (starch and soluble sugars) in leaves and branches of 82 tropical tree-species. Species are from six sites in the Amazon Basin with contrasting mean annual precipitation and dry season length .
Research sample	Sampling was focused on the most dominant canopy species in terms of basal area, with the total number of species sampled at each site ranging from 9 to 31. The organisms studied are canopy trees from 82 species, 63 genera and 29 families (Supplementary Table 1). Species-level identification of all trees sampled is based on botanical vouchers previously collected and deposited in Amazon state herbaria (AMAZ, CUZ, HOXA, INPA, UFACPZ, USZ) by RAINFOR partners. Trees are tagged and identifications were obtained from the ForestPlots.net database (https://www.forestplots.net/; KEN, FEC, TAM, ALP and SUC) and from collaborator databases for MAN.
Sampling strategy	Our aim was to collect the most representative species in each site based on the total basal area. Inventory data from the RAINFOR network was used to calculate the basal area in Ken, Fec, Tam, Alp and Suc, and from collaborator databases for Man. The sampling effort varied from 9 to 31 species, accounting for between 12% to 47% of the total basal area in each site (Tab. 1). The sites which we sampled less than 40% of the total basal area (Man, Alp and Suc) are hyperdiverse forests and lack clear species dominance (i.e. where there are few species contributing to most of the total basal area of the plot). The high diversity, difficult logistics and high costs of these analysis impose strong constraints on the number of species that can be realistically sampled. The sample sizes used for our NSC measurements are in line with those used in other tropical sites and have been shown to be sufficient to capture community-level distributions of functional traits. For example, Barros et al. (2019) sampled hydaraulic traits for 17 species and showed that the community-level mean values did not change when extending this analysis to a total of 28 trees. Thus, our mean NSC values are likely to well represent the evaluated communities. Whenever possible, we sampled trees of similar sizes within a same species and site (DBH > 20 cm), and we also avoided sampling shaded trees to avoid the confounding effects of differential light regimes.
Data collection	Leaf and branch samples were obtained by a tree climber. To minimise effects of diurnal changes in NSC concentrations, samples were obtained before sunrise in all sites, except MAN. In MAN, branches and leaves were obtained just after sunrise and always before 8 a.m.
Timing and spatial scale	Plant material for NSC analyses was collected in all sites during the wetter months. For the four sites with more marked seasonality (KEN, FEC, MAN and TAM), plant material for NSC analyses was also collected in the period of lowest monthly precipitation as detailed in Extended Data Fig. 1 and Tab. 1.
Data exclusions	Species collected in the wet season that did not keep their leaves during the dry season were excluded from all analysis to avoid potential biases due to different phenological strategies.
Reproducibility	Considering the constraints related to sample collection, transport, exportation and analyses, the experiments new analyses could reach slightly different values. NSC concentrations and leaf water potential may vary depending on climatic conditions, also NSC concentrations may vary depending on the method used for quantification and the laboratory where the analyses were performed. Despite the technical challenge, all information is provided in the Methods section to allow the experiments to be reproduced. Sample collection was performed only once for each site and season as described in Methods. Species collected in the wet season that did not keep their leaves during the dry season were excluded from all analysis to avoid potential biases due to different phenological strategies. The NSC content of branches and leaves was replicated twice, and the mean used as the sample NSC value for further analysis.
Randomization	This is not relevant for our study. Tree branches were collected when they met our criteria, as follow, have the leaves completely expanded, without liana infestation or injures and not shaded. In the laboratory, the samples of each tree were identified with a numerical code instead of the scientific name, and the NSC analyses carried out randomly.
Blinding	Blinding was not relevant for our study, since knowing the identity of the trees we are sampling was important.

Did the study involve field work? ☒ Yes ☐ No

Field work, collection and transport

Field conditions	Parameters that were relevant at the time of sampling (precipitation and EVI) are provided in Supplementary Fig. 1 and 5. Precipitation data is from worldclim.org/data/index.html, and the adjusts MODIS EVI product is from doi:10.5281/ZENODO.3159488.

	Data where extract for the coordinates that are show in Extended Data Table 1.
Location	Samples where collected in permanent forest plots<br>Ken - Ascensión de Guarayos, Santa Cruz, Bolivia 16°1'S, 62°43'W<br>Fec - Senador Guiomard, Acre, Brazil 10°4'S, 67°37'W<br>Man - Manaus, Amazonas, Brazil 2°36'S, 60°12'W<br>Tam - Puerto Maldonado, Madre de Dios, Peru 12°49'S, 69°16'W<br>Alp - Iquitos, Maynas, Peru 3°56'S, 73°25'W<br>Suc - Iquitos, Maynas, Peru 3°15'S, 72°54'W
Access & import/export	Collection permits for all sites except Man, were granted in the name of David R. Galbraith/Tremor Project. For Man was granted to Laura S. Borma.<br>The permits for sample collection and exportation in each site was concede for the responsible authorities as follow:<br>- Tam - Dirección de Gestión de las Áreas Naturales Protegidas (SERNANP). nº 039-2016-SERNANP-RNTAMB-PRD, Date: 08/09/2016;<br>- Alp - Dirección de Gestión de las Áreas Naturales Protegidas (SERNANP). nº 073-2017-SERNANP-RNAM-J, Date: 18/11/2016;<br>- Suc - Gestión Sostenible del Patrimonio Forestal y de Fauna Silvestre (SERFOR). nº121-2016-GGR-ARA-DEFFS-DER, Date: 29/11/2016;<br>- Ken - Ministerio de Medio Ambiente y Agua - Viceministerio de Medio Ambiente, Biodiversidad y Cambios Climáticos. CAR-MMAYA/VMABCCGDF/DGBAP/MEY nº 0198/2017, Date: 27/03/2017;<br>- Fec - Instituto Chico Mendes de Conservação da Biodiversidade (ICMBio), Sistema de Autorização e Informação em Biodiversidade (SISBIO) - Número: 57821-1, Date: 07/03/2017;<br>- Man - Instituto Chico Mendes de Conservação da Biodiversidade (ICMBio). Sistema de Autorização e Informação em Biodiversidade (SISBIO) - Número: 44404-4, Date: 15/06/2015.<br><br>Permits for import samples to Brasil from Peru and Bolivia was concede by Ministério da Agricultura, Pecuária e Abastecimento. Oficio nº 334/2016/SSV-SP - MAPA
Disturbance	Tree's canopies were accessed by climbers using climbing equipment, which avoids damage to the tree trunk. Terminal branches were cut with pole prune scissors and there were no disturbances at the plot.

Reporting for specific materials, systems and methods

We require information from authors about some types of materials, experimental systems and methods used in many studies. Here, indicate whether each material, system or method listed is relevant to your study. If you are not sure if a list item applies to your research, read the appropriate section before selecting a response.

Materials & experimental systems

n/a	Involved in the study
☒	☐ Antibodies
☒	☐ Eukaryotic cell lines
☒	☐ Palaeontology and archaeology
☒	☐ Animals and other organisms
☒	☐ Human research participants
☒	☐ Clinical data
☒	☐ Dual use research of concern

Methods

n/a	Involved in the study
☒	☐ ChIP-seq
☒	☐ Flow cytometry
☒	☐ MRI-based neuroimaging
